# Genomic prediction-aided incorporation of genetic resources into elite breeding: lessons from a collaborative multiparental design in flint maize

**DOI:** 10.1007/s00122-025-05034-3

**Published:** 2025-10-01

**Authors:** Dimitri Sanchez, Sarah Ben Sadoun, Tristan Mary-Huard, Cyril Bauland, Carine Palaffre, Bernard Lagardère, Delphine Madur, Valérie Combes, Stéphane Melkior, Laurent Bettinger, Alain Murigneux, Laurence Moreau, Alain Charcosset

**Affiliations:** 1 Université Paris-Saclay, INRAE, CNRS, AgroParisTech, Génétique Quantitative et Evolution – Le Moulon, 91190 Gif-sur-Yvette, France; 2 LIDEA FRANCE, Avenue Gaston Phoebus, 64230 Lescar, France; 3 Université Paris-Saclay, AgroParisTech, INRAE, UMR MIA-Paris Saclay, 91120 Palaiseau, France; 4 UE 0394 SMH, INRAE, 2297 Route de L’INRA, 40390 Saint-Martin-de-Hinx, France; 5 RAGT2n, 12510 Druelle, France; 6 Limagrain Europe, 28 Route d’Ennezat, 63720 Chappes, France

## Abstract

**Key message:**

A public private cooperative mating design between elite maize inbred lines and diversity donors shows that genomic prediction holds great promise to improve the use of genetic resources.

**Abstract:**

Genetic diversity is essential for plant breeding, enabling long-term gains and adaptation to climate change and new agronomical practices. Breeders can access diverse genetic resources to enhance elite germplasm and introduce new favorable variations. The limited performance of genetic resources may hamper their use. To overcome this, a bridging population can be implemented to evaluate and select progenies from crosses between diversity donors and elite lines before their introduction in breeding programs. The choice of such crosses can be dealt with the usefulness criterion (UC), which determines its ability to produce transgressive individuals. This paper investigates the use of genome-wide marker effects to predict (i) the performance of individuals derived from crosses between donors and elite lines and (ii) the UC of crosses not observed yet. It also compares donor introduction strategies based on the UC or the H criterion, which considers the genome-wide donor–elite complementarity. We used a flint maize collaborative multi-parental BC1-S2 population, consisting in materials from six breeding companies and one public institute crossed to different donors. The 20 crosses had contrasted means and genetic variances, and most of them presented transgressive individuals above the elite parent. Results emphasize the importance of half-siblings derived from the elite line parent of the predicted cross to efficiently predict progeny performances or the UC. They also showed that using the H criterion appears promising to select iteratively donors that best complement initial elite materials. The paper concludes with guidelines for implementing a bridging population using genome-wide marker-based predictions.

**Supplementary Information:**

The online version contains supplementary material available at 10.1007/s00122-025-05034-3.

## Introduction

The success of a plant breeding program relies on its ability to release competitive varieties in the short term while guaranteeing long-term genetic gain. To achieve this goal, preserving the genetic diversity in the breeding program is needed, as it is a key determinant of trait genetic variance, which controls the expected response to selection per generation (Lush 1937). Several studies have shown that over breeding cycles, genetic progress is often accompanied by a narrowing of the elite germplasm genetic diversity since new lines and varieties are derived mainly from a limited number of founder lines (Reif et al. 2005; Mikel and Dudley 2006; Rauf et al. 2010). For instance, Allier et al. (2019b) documented a loss of genetic diversity over time in a European maize breeding program. Recent breeding methods, such as genomic selection which is now used in routine, may accelerate genetic diversity depletion by favoring the selection of highly related individuals (Jannink 2010; Rutkoski et al. 2015; Lin et al. 2016). Beyond reducing the genetic gain, low genetic diversity may hamper breeding potential to address new selection objectives related to climate change and evolution in agronomical practices (Fess et al. 2011; McCouch et al. 2013; Mickelbart et al. 2015). The reduction of genetic gain can be delayed, to some extent, by the use of efficient breeding program diversity management (Allier et al. 2020a). However, external diversity sources are needed to bring back genetic variation and compensate for the negative impact of selection on genetic diversity (Wray and Goddard 1994; Woolliams et al. 2015; Allier et al. 2020a).

Plant breeders can access a large range of diversity sources to enhance the potential of their programs. Their incorporation into a specific program can be more or less challenging according to their adaptation to the environment targeted by the program and their performance (Sanchez et al. 2024). The closest sources to be considered are competitor materials from programs with the same target environment. Varieties under the International Union for the Protection of New Varieties of Plants (UPOV) can be used straightforwardly for selection of inbred varieties. Their use is more challenging for hybrid varieties due to the organization of breeding programs into heterotic groups, necessitating for instance to implement reverse breeding (Smith et al. 2008). Commercial exchange of elite parental lines can be an appealing alternative in this case. More distant materials may also be examined, such as ex-Plant Variety Protection (PVP) lines for maize in the USA. These lines have a 20-year performance gap with the current elite material, but they present original interesting genetic variability which fits into established heterotic groups (Mikel and Dudley 2006; Kurtz et al. 2016). Landraces and historical lines preserved thanks to an intense effort of collection and maintenance have been sown to be sources of original alleles, particularly for adaptation to abiotic or biotic stresses (Dwivedi et al. 2016; Romero Navarro et al. 2017). The interest in landraces is currently renewed by the possibility of developing inbred lines using doubled-haploid technology (Strigens et al. 2013; Böhm et al. 2017; Brauner et al. 2018; Mayer et al. 2020).

An efficient diversity enrichment strategy must identify the most interesting genetic resources, i.e., those which carry favorable alleles not yet, or no longer, present in the breeding program and ensure the incorporation of these alleles. For monogenic or oligogenic traits, i.e., traits determined by some major genes, the integration of favorable alleles can be performed thanks to backcross or gene pyramiding approaches (Hospital and Charcosset 1997; Peng et al. 2014; Han et al. 2017). For quantitative performance traits (e.g., grain yield in maize) determined by numerous genomic regions with minor effects, selecting the appropriate donor lines is more complicated. In this case, the most promising crosses between candidate diversity donors and elite lines are those with the highest usefulness criterion (UC), corresponding to the expected mean performance of the cross-progeny after selection (Schnell and Utz 1976). The UC of a cross is defined as a sum of the expected mean progeny performance $\mu$ and the expected genetic gain ($ih\sigma$) that can be achieved by selecting within the progeny: $\mu +ih\sigma$ where $i$ is the selection intensity, $h$ is the prediction accuracy, and $\sigma$ is the progeny genetic standard deviation. $h$ can be assumed to be one when selection occurs on genotypic effects estimated with sufficient information (Zhong and Jannink 2007). This criterion privileges the crosses most likely to generate positive transgressive individuals. Using UC to compare donor x elite crosses is particularly pertinent because large differences in progeny variances between crosses are expected (Mohammadi et al. 2015; Lado et al. 2017; Yao et al. 2018). This theoretical expectation was supported by observing such differences in multi-parental maize populations, including populations derived from crosses between elite lines and genetic resources (Lehermeier et al. 2014; Sanchez et al. 2024).

Determining the UC of a cross before it is made requires predicting the value of the mean and variance of its progenies (Oget-Ebrad et al. 2024). If the parental performances are known, the progeny mean can be easily derived for different crossing schemes by weighted parental performances (Utz et al. 2001; Bernardo 2014). The attempts to predict the progeny variance using morphological or pedigree information showed poor results reviewed in (Mohammadi et al. 2015). The genetic distance between the parents, computed thanks to marker information, was also revealed to be a poor predictor of progeny variance (Bohn et al. 1999; Hung et al. 2012; Beckett et al. 2019). The availability of low-cost high-density genotyping opened the way for using genome-wide marker effects to predict the UC values. Mohammadi et al. (2015) and Bernardo (2014) proposed simulating in silico progenies whose values were determined thanks to marker effects estimated from a model calibrated with the phenotypes and the genotypes of the parental lines to forecast the progeny variance of potential crosses. This method has been tested on bi-parental populations in barley (Tiede et al. 2015; Neyhart and Smith 2019), wheat (Lado et al. 2017; Yao et al. 2018) and maize (Adeyemo and Bernardo 2019). It performed inconstantly but has been proven more efficient than the previous methods. Lehermeier et al. (2017) derived algebraic formulas to compute the progeny variances of bi-parental crosses using marker effect estimations, recombination rates between markers and linkage disequilibrium in parental lines. This approach avoids the progeny simulation step, which can be computationally time-consuming when the number of potential crosses increases. Allier et al. (2019a) extended the algebraic formulas to multi-parental crosses. The efficiency of this approach was validated with simulations and experimental results for some traits in wheat (Oget-Ebrad et al. 2024). To our knowledge, no study has compared progeny variances calculated analytically and those estimated from progeny phenotypic evaluations in the context of donor introduction.

Using marker effects to predict the progeny variance raises the question of defining a relevant set of individuals (training set, TS). This question is primordial in genomic selection (GS) that is now commonly used in breeding programs (Heslot et al. 2015; Crossa et al. 2017). To maximize the efficiency of the GS methods, a suitable TS should maximize the relationship with the predicted individuals (prediction set, PS) and minimize the relationship within the TS (Isidro Y Sánchez and Akdemir 2021). For crosses involving diversity donors, TS must be able to estimate the effects of donor alleles absent or in low frequency in the elite lines. Allier et al. (2020a) suggested using a large panel of lines covering a continuum from founders of current breeding pools to elite lines. However, Adeyemo and Bernardo (2019) indicated that a calibration based on a diverse panel of maize inbreds was ineffective in predicting progeny variances of crosses. This conclusion was supported by the experimental evaluation of only eight crosses because of logistic costs. Guidelines to define a TS adapted to predict the UCs of donor x elite crosses to facilitate donor introductions remain to be established.

The UC allows one to detect the most promising crosses, but it does not explicitly give access to the complementary of potential parents along the genome. Allier et al. (2020a) recommended considering this complementary in choosing diversity donors to guarantee long-term genetic gain. The authors introduced the H criterion adapted from the optimal haploid value [OHV, (Daetwyler et al. 2015)] and the optimal population value [OPV, (Goiffon et al. 2017)]. This criterion relies on identifying favorable donor haplotypes absent from the elite population and prioritizing the introduction of donors which accumulate those haplotypes. In Allier et al. (2020b) the UC and the H criterion were positively correlated, but further investigations on the interest of their potential combined implementation are needed.

After identifying the most promising donors, a suitable diversity introduction strategy must be established to avoid a negative impact on the genetic gain in the breeding program. For donors suffering from agronomical defaults or a performance gap compared to the elite material, a direct introduction can lead to a drastic loss of genetic gain and threaten the economic sustainability of the breeding program (Allier et al. 2020a). Simmonds (1993) proposed to improve the performance of individuals derived from donor x elite crosses in a bridging population before an actual introduction into the breeding program. Simulation studies showed that this strategy efficiently preserved genetic diversity and improved long-term gains (Allier et al. 2020a; Vanavermaete et al. 2021; Sanchez et al. 2023). The initial motivation of this approach was to reduce the performance gap while revealing original favorable alleles carried by the donors. It is also a way to improve the calibration of GS models used to predict the interest of new crosses and to detect donors carrying genomic segments superior to those currently segregating in the elite pool. For a breeder with limited resources, implementing a bridging population competes with conducting its elite breeding programs. Using GS approaches to predict the value of bridging individuals and identify the best individuals to introduce may help to increase the efficiency of a bridging population.

In this paper, our objectives were to (i) evaluate experimentally the interest of GS to predict the values of individuals derived from donor x elite crosses for different traits, (ii) evaluate the efficiency of the method proposed by (Lehermeier et al. 2017) to predict the UC of unobserved crosses for performance traits and (iii) compare the use of the H criterion and the UC to prioritize additional crosses not yet made. We considered to do so a maize cooperative multi-parental population of 20 BC1 connected families derived from crosses between diversity donor lines chosen for their originality and initial performance and elite recipient lines from different private partners. This population had revealed large differences in means and variances in a preliminary study (Sanchez et al. 2024). We extended here its evaluation with additional trials and considered mid-density genotypic information to address above-mentioned questions. The family structure of this population made it possible to address the impact of the relationship level between the TS and the predicted individuals on the efficiency of genomic-based predictions.

## Material and methods

### Plant material, phenotypic and genotyping data

Our study relies on a collaborative multi-parental population composed of 1,174 flint maize BC1S2 individuals derived from twenty biparental crosses between seven elite recipient lines and nine diversity donor lines chosen based on their originality, performance and limited agronomic defaults (Sanchez et al. 2024).

**Fig. 1:**
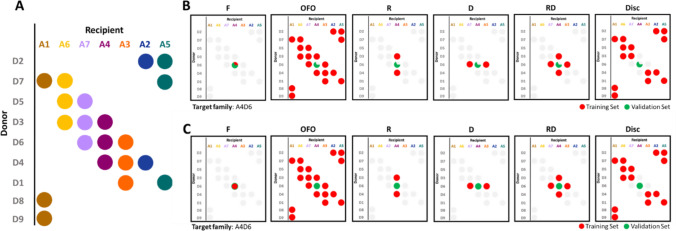
Incomplete crossing design between the recipient and donor parental lines (**A**) and illustration (with A4D6 as target family) of training sets (TS) considered for the prediction of the progeny performances (**B**) and for the prediction of mean and variance values of a cross (**C**). F: within-family prediction, OFO: One-family-out prediction, R: The TS involved individuals derived from crosses with the same recipient than the target family, D: The TS involved individuals derived from crosses with the same donor, RD: The TS involved individuals derived from crosses with the same donor or recipient, Disc: The TS involved individuals derived from different donor and recipient lines. (**B**) Two-third of the individuals of the target family were sampled for use as validation set (VS), and their values were predicted thanks to the different TS types (100 repetitions). (**C**) The individuals of the TS are used to estimate marker effects to predict the mean and variance of the target family

An incomplete mating design was applied to cross the recipient (R) and donor (D) lines (Fig. 1). Each F1 single-cross hybrid was backcrossed with the recipient line to obtain BC1 populations. For each DxR cross, BC1 plants were self-pollinated during two generations (single seed descent process) to get BC1S2 individuals. The targeted number of BC1S2 per cross was 60. It was determined considering a fixed global effort of 180 individuals per partner and the need to compromise between (i) individual population sizes high enough to estimate variances and (ii) the need to establish connections between populations through the use of donors common to several recipient lines. Actual number of individuals per cross was 55.25 on average and varied from 38 to 66. To perform test cross phenotypic evaluation, each BC1S2 plant was self-pollinated to generate a BC1S2:3 progeny. Hybrids were produced by crossing the BC1S2:3 families and the dent tester line associated with their recipient parental line. In the following, hybrids derived from the same DxR cross are considered as a family.

Hybrids were evaluated in seven different trials in 2019 (Blois, Loir-et-Cher, France; Saint-Martin de Hinx, Landes, France; Villers, Pas-de-Calais, France) and 2021 (Réclainville, Eure-et-Loir, France; Villampuy, Eure-et-Loir, France; Selommes, Loir-et-Cher, France; Einbeck, Niedersachsen, Germany). These trials were named Blo19, Smh19, Vil19, Rec21, Vily21, Sel21 and Ein21. Each trial comprised elementary two-row plots with an average area of 9.8 m^2^ and an average plant density of 10 plants.m^−2^. In 2019, 1,512 plots were phenotyped in each trial. In 2021, the total size differed between trials: 1,404 plots were evaluated in Rec21, Vily21 and Sel21 and 1,560 plots in Ein21. Trial structure and preliminary statistical analyses of 2021 trials are equivalent to that presented in Sanchez et al. (2024) for 2019 trials and are recalled in Supplementary 2.2 Material and methods.

Male and female flowering times (FLOM and FLOF in days after planting), grain yield at 15% moisture (GY in 0.1t.ha^−1^), grain humidity at harvest (H2O in %) and plant height (PH in cm) were evaluated. FLOM was not recorded in Sel21, and PH was not measured in Vil19 and Sel21. FLOM and FLOF were scored as the day at which 50% of the plants exhibited anthers or silks, respectively. Anthesis-silking interval (ASI) was computed as the difference in days between FLOM and FLOF. A commercial grain yield index (YI, in 0.1t.ha^−1^) was calculated after field spatial correction (Supplementary Material and Methods):

$$Y{I}_{m}=G{Y}_{m}+2.5*(H{20}_{\text{ADEVEY}}-H2{O}_{m})$$

where $G{Y}_{m}$ and $H2{O}_{m}$ are the corrected grain yield and humidity values of plot $m$ and $H{20}_{\text{ADEVEY}}$ is the trial mean value of ADEVEY grain humidity. This index corresponds to the one usually considered for variety registration in France.

BC1S2 individuals and their parents were genotyped using a customized Maize Illumina 25k single nucleotide polymorphism (SNP) XT array, used as marker information in our study. After filtering monomorphic markers, 19,311 SNPs were retained. Marker genetic positions were determined from their physical position using a spline-smoothing interpolation procedure following Bauer et al. (2013) and the consensus flint maize map of Giraud et al. (2014).

### Evaluation of the mean performances and additive genetic variances of the hybrid families

All parameter estimations and genomic predictions were performed on the adjusted means of the hybrids over the seven trials after spatial correction, noted $Y$ (Supplementary Material and Methods). A mixed linear model was fitted to estimate the mean performance (${\mu }_{k}$) and additive genetic variance (${\sigma }_{Ak}^{2}$) of each family $k$:

$${Y}_{hk}={\mu }_{k}+ {A}_{hk}+{E}_{hk},\quad {{\varvec{A}}}_{{\varvec{k}}}\sim N\left(0,{{{\varvec{K}}}_{{\varvec{k}}}\sigma }_{Ak}^{2}\right), \ {\varvec{E}}\sim N\left(0,{{\varvec{I}}\sigma }_{E}^{2}\right) \text{ind}, {\varvec{A}}\perp {\varvec{E}} (M1)$$

where ${Y}_{hk}$ is the adjusted mean of the hybrid $h$ derived from the cross $k$. ${\mu }_{k}$ is the intercept, ${A}_{hk}$ is a random additive genetic effect, and ${E}_{hk}$ is the error term. ${{\varvec{K}}}_{{\varvec{k}}}$ is a marker-based additive kinship matrix specific to the family $k$. We performed independent estimations of these parameters from one family to another to account for family-specific allele frequencies.

For performance traits (GY and YI), the interest of each family was evaluated by computing its usefulness criterion ($U{C}_{k}$):

$$U{C}_{k}= {\mu }_{k}+ih{\sigma }_{Ak}^{2}$$

where $h$ is the prediction accuracy and $i$ is the selection intensity. The prediction accuracy was set to one, as would be the case when selecting directly on genetic effects (Zhong and Jannink 2007). We used an intensity of selection equal to 2.07, corresponding to a selection rate of 5%.

### Prediction of the progeny values and the usefulness criterion of a family

We investigated the effect of the composition of training sets to (i) predict the values of hybrids derived from a specific cross, i.e., within a given family, and (ii) predict the usefulness criteria for new crosses (Fig. 1).

#### Evaluation of the within-family predictive abilities

As a preliminary step, we used a GBLUP model to perform cross-validations in each family to evaluate the within-family predictive abilities (PAs). This step allowed us to check the efficiency of the prediction model and to define a baseline PA value for each family before performing inter-family predictions (see next section). To compare the efficiency of within-family and inter-family predictions, we worked with validation sets (VS) common to both prediction types. For each family, a VS was composed of a random sample of two-thirds of the hybrids (Fig. 1). For the within-family predictions, their phenotypic values were predicted using the following GBLUP model calibrated with the remaining third of the individuals (training set, TS):

$${\varvec{y}}={1}_{{{\varvec{n}}}_{{\varvec{T}}{\varvec{S}}}}.\mu +\boldsymbol{ }{\varvec{Z}}{\varvec{A}}+{\varvec{E}} , \quad {\varvec{A}}\sim N\left(0,{{\varvec{K}}\sigma }_{A}^{2}\right),\text{ }\boldsymbol{ }{\varvec{E}}\sim N\left(0,{{\varvec{I}}\sigma }_{E}^{2}\right),\text{ } {\varvec{A}}\perp {\varvec{E}} (M2)$$

where ${\varvec{y}}$ is the vector of ls-means of the ${n}_{TS}$ individuals of the TS, ${1}_{{{\varvec{n}}}_{{\varvec{T}}{\varvec{S}}}}$ is a vector of ${n}_{TS}$ ones, $\mu$ is the intercept, ${\varvec{Z}}$ is an incidence matrix of dimension $\left[{n}_{TS} \times n\right]$ with $n={n}_{TS}+{n}_{VS}$ where ${n}_{VS}$ is the number of individuals in the VS**, A** is the vector of random additive genetic effects, and ${\varvec{E}}$ is the vector of error terms. ${\varvec{K}}$ is a marker-based kinship matrix among the $n$ individuals (see Estimation of marker-based kinship matrices section).

This process was repeated 100 times. For each repetition, the PA was computed as the correlation between the observed phenotypic values (ls-means) and the predicted values for the individuals of the VS. The within-family average predictive ability corresponds to the average of the 100 PAs and is labeled F in the following.

M1 and M2 models were fitted using the MM4LMM R package (Laporte et al. 2022).

#### Evaluation of predictive abilities of different training sets

We first considered training sets with no hybrid from the target family. We performed “one-family-out” predictions (OFO), using for each target family the hybrids of all other families as TS. To explore the impact of the relatedness between individuals in the TS and the VS, we also tested four alternative TS compositions by subsetting families in the OFO TS based on their relationship level with the target family. These subsets were labeled R, D, RD and Disc (Fig. 1). R comprised only the hybrids produced from the same recipient as the predicted hybrids. D contained only the hybrids derived from the same donor. RD was the addition of the individuals of R and D. Disc gathered the hybrids derived from crosses disconnected from the target family (different recipient and donor parents). For each target family, we calibrated the GBLUP model (M2) using the different TS types (OFO, R, D, RD and Disc) to predict the individuals of the VSs sampled for the within-family prediction evaluation (100 repetitions). We computed the mean PA for each family and each TS type. Note that for prediction sets that involve several testers (OFO, D, RD and Disc) dominance effects could have been added to model M2 to account for variation within dent parental lines. We restricted our analysis to additive effects to be able to use the same model for all calibration set types (including R and single population TS), and because literature highlights a clear preponderance of additive effects in inter-heterotic group crosses, particularly for flint x dent hybrids (see Lorenzi et al 2022).

The same prediction procedure was followed by adding to the TS the individuals of the target family not used in the VS (one-third of the family) to investigate the interest of phenotyping part of the family.

#### Ability of different training sets to predict the usefulness criterion

We explored the interest of our design to predict the UCs of new recipient x donor crosses for which no progeny family was produced yet. To address this question, we predicted the mean performance and the additive genetic variance of each cross using the different TS types presented in the previous section (OFO, R, D, RD and Disc, Fig. 1). We also estimated as a benchmark the same parameters using all individuals of the predicted cross (within family prediction, F). To achieve these predictions, we followed the method presented by Lehermeier et al. (2017) and Allier et al. (2019a). For each cross and each TS, we estimated marker effects using the following Bayesian ridge regression (BRR) model:

$${\varvec{y}}={{\varvec{X}}}_{{\varvec{T}}{\varvec{S}}}{\varvec{\beta}}+{\varvec{E}} \left(M3\right)$$

$${\varvec{\beta}}|{\sigma }_{ \beta }^{2}\sim N\left(0,{{\varvec{I}}\sigma }_{ \beta }^{2}\right), \ {\varvec{E}}|{\sigma }_{ E}^{2}\sim N\left(0,{{\varvec{I}}\sigma }_{E}^{2}\right)$$

$${\sigma }_{ \beta }^{2} \sim \text{Scale}-\text{inv}-{\chi}^{2}\left({\nu }_{\beta },{\tau }_{\beta }\right), \text{ } {\sigma }_{ E}^{2} \sim \text{Scale}-\text{inv}-{\chi}^{2} ({\nu }_{E},{\tau }_{E})$$

where ${\varvec{y}}$ is the vector of ls-means of the ${n}_{TS}$ individuals of the training set (TS), ${{\varvec{X}}}_{{\varvec{T}}{\varvec{S}}}$ is the $\left[{n}_{TS} \times (M+1)\right]$ genotyping matrix coded in 0 or 2 with $M$ the number of markers**,** ${\varvec{\beta}}$ is the vector of random marker effects (and intercept), and ${\varvec{E}}$ is the vector of error terms. M3 was fitted with the BGLR R package (Pérez and de los Campos 2014). Hyperparameters (${\nu }_{\beta },{\tau }_{\beta }, {\nu }_{E}$ and ${\tau }_{E}$) for the scaled inverse-${\chi}^{2}$ prior distributions were chosen using the default rules in BGLR with an a priori assumption of 50% of the phenotypic variance explained by the markers. We used 20,000 iterations, where the first 5000 were discarded as burn-in. One-fifth of the postburn-in iterations were kept for posterior inference resulting in a total of $S=3000$ samples. The vector of posterior mean marker effects ($\widehat{{\varvec{\beta}}})$ was computed as the mean of marker effects estimated in the $S$ samples. Only additive effects were considered for the reasons given above in the “Evaluation of predictive abilities of different training sets” section.

For each cross and each TS, the mean value of the cross was predicted from the parental line values considering the expected parental genome proportions after the backcrossing step:

$$\widehat{{\mu }_{k}}= \frac{3}{4}{{\varvec{x}}}_{{\varvec{R}}}\widehat{{\varvec{\beta}}}+\boldsymbol{ }\frac{1}{4}{{\varvec{x}}}_{{\varvec{D}}}\widehat{{\varvec{\beta}}}$$

where ${{\varvec{x}}}_{{\varvec{R}}}$ and ${{\varvec{x}}}_{{\varvec{D}}}$ are the genotyping vectors of the recipient and the donor lines.

To estimate progeny variance, Lehermeier et al. (2017) proposed two alternative methods: the “variance of posterior means” (VPM) and the “posterior mean variance” (PMV). The VPM method estimated progeny variance using the vector of posterior means of marker effects ($\widehat{{\varvec{\beta}}})$:

$${\sigma }_{A,k}^{{2}^{\left[VPM\right]}}=\boldsymbol{ }{\widehat{{\varvec{\beta}}}}^{\boldsymbol{^{\prime}}}{\varvec{\Sigma}}\widehat{{\varvec{\beta}}}$$

where ${\varvec{\Sigma}}$ is the variance–covariance matrix between the markers in the progeny of dimension $[M \times M]$ (computation detailed below).

The PMV method relied on the computation of the progeny variance in each MCMC sample as follows:

$${\sigma }_{A,ks}^{2}={{\varvec{\beta}}}^{({\varvec{s}})\boldsymbol{^{\prime}}}{\varvec{\Sigma}}{{\varvec{\beta}}}^{({\varvec{s}})}$$

where ${{\varvec{\beta}}}^{({\varvec{s}})}$ is the *s*th thinned postburn-in sample ($s \in [1:S]$). The PMV progeny variance was derived as the posterior mean of variances from all samples:

$${\sigma }_{A,k}^{{2}^{\left[\text{PMV}\right]}}=\boldsymbol{ }\frac{1}{S}\sum_{s=1}^{S}{\sigma }_{A,ks}^{2}$$

${\varvec{\Sigma}}$ was derived from the parental genotypes and the recombination rate frequencies. For a population of lines derived from BC1 individuals, the covariance between the marker $i$ and $j$ was expressed as:

$${\Sigma }_{ij}={D}_{jl}^{P}(1-2{c}_{ij})(3-2{c}_{ij})$$

${D}_{jl}^{p}$ denotes the linkage disequilibrium in the pair of parental lines between the alleles of the markers $i$ and $j$. ${D}_{jl}^{p}$ is either 0 if both parental lines show the same alleles at one or both markers, or 0.25 or − 0.25 depending on the linkage phase of the parents. ${D}_{jl}^{p}$ can be computed as ${D}_{jl}^{p}=\frac{1}{16}{\left[\left({{\varvec{x}}}_{{\varvec{R}}}-{{\varvec{x}}}_{{\varvec{D}}}\right){\left({{\varvec{x}}}_{{\varvec{R}}}-{{\varvec{x}}}_{{\varvec{D}}}\right)}^{\boldsymbol{^{\prime}}}\right]}_{ij}$. ${c}_{ij}$ is the expected recombination frequency between both markers. This frequency increases with increasing the number of meiosis. For a BC1S2 population, ${c}_{ij}$ is equal to:

$${c}_{ij}= \frac{2{c}_{ij}^{1}}{1+ 2{c}_{ij}^{1}}\left(1-\frac{1}{4}{\left(1-2{c}_{ij}^{1}\right)}^{2}\right)+ \frac{1}{2}{\left(\frac{1}{2}\left(1-2{c}_{ij}^{1}\right)\right)}^{2}$$

where ${c}_{ij}^{1}$ is the expected recombination rate in a DH population derived from a F1 cross between both parents obtained from the genetic distance between both markers ${d}_{ij}$ in Morgan as ${c}_{ij}^{1}= \frac{1}{2}(1-{e}^{-2{d}_{ij}})$ (Haldane 1919).

For each cross and each TS, two UC estimations were computed using the VPM or PMV variance progeny predictions:

$$U{C}_{k}^{\left[\text{VPM}\right]}=\widehat{{\mu }_{k}}+i*{\sigma }_{A,k}^{{2}^{\left[\text{VPM}\right]}} \text{ and } U{C}_{k}^{\left[\text{PMV}\right]}=\widehat{{\mu }_{k}}+i*{\sigma }_{A,k}^{{2}^{\left[\text{PMV}\right]}}$$

For the prediction of the mean performance, we compared the method described above with a prediction obtained by adding the corresponding parental effects estimated from the following model fitted using the OFO TS individual observations:

$${Y}_{ijh}= \mu + {{\gamma }_{i}+\rho }_{j}+{E}_{ijh} ,\quad {\varvec{E}}\sim N\left(0,{{\varvec{I}}\sigma }_{E}^{2}\right) \text{ind} (M4)$$

where ${Y}_{ijh}$ is the adjusted means of the hybrid $h$ derived from the donor $i$ and the recipient $j$. $\mu$ is the intercept, ${\gamma }_{i}$ is the fixed effect of the donor, ${\rho }_{j}$ is the fixed effect of the recipient, and ${E}_{hk}$ is the error term. In this case, the mean performance of the predicted cross was computed by adding the intercept value and estimated effects of the parents involved in the cross.

To compare the performance of mean, variance and UC predictions between the different TSs, we computed the correlation between predicted and observed values for the twenty crosses and the Normalized Root Mean Square Error (NRMSE). NRMSE was defined as:

$$\text{NRMSE}=\frac{\text{RMSE}}{\left({x}_{\text{obs},\text{max}}- {x}_{\text{obs},\text{min}} \right)} ,\text{ with} \text{ RMSE}=\sqrt{\frac{1}{20}\sum_{i=1}^{20}{\left({x}_{\text{pred},i}-{x}_{\text{obs},i}\right)}^{2}}$$

where ${x}_{\text{pred}}$ and ${x}_{\text{obs}}$ were the predicted or observed values (for mean, variance or UC).

##### Estimation of marker-based kinship matrices

The kinship matrices used in spatial correction models and the GBLUP models were computed based on the BC1S2 individual genotypes, according to the Natural and Orthogonal Interaction Approach (NOIA, 60), as recommended by Vitezica et al. (2017). For a given individual $i$, at a given marker $j$, coefficients were calculated using genotypic frequencies as follows:

$${h}_{{A}_{ij}}=\left\{\begin{array}{l}-(-{p}_{B{b}_{j}}-2{p}_{b{b}_{j}})\\ -(1-{p}_{B{b}_{j}}-2{p}_{b{b}_{j}})\\ -(2-{p}_{B{b}_{j}}-2{p}_{b{b}_{j}})\end{array}\right.\text{ for genotypes}\left\{\begin{array}{l}BB\\ Bb\\ bb\end{array}\right.$$

where ${p}_{B{B}_{m}}, {{p}_{Bb}}_{m}$ and ${p}_{b{b}_{m}}$ are the frequencies of genotypes BB, Bb and bb in the relevant subset of $n$ individuals (BC1S2 derived from all crosses for spatial corrections or from the crosses involved in training and validation sets for GBLUP models).

Additive kinship matrices were obtained with the formula:

$$K = \frac{{H_{A} H^{\prime }_{A} }}{{tr\left( {H_{A} H^{\prime }_{A} } \right)/n}} {\text{ with }} H_{A} = \left( {\begin{array}{*{20}c} {h_{{A_{11} }} \cdots h_{{A_{1m} }} } \\ { \vdots { } \cdots { } \vdots } \\ {h_{{A_{n1} }} \cdots h_{{A_{nm} }} } \\ \end{array} } \right)$$

where $m$ is the number of polymorphic markers in the subset of individuals.

##### Ranking of new donors to enrich the recipient lines

In our design, each recipient line was crossed to only a fraction of the donor lines (two or three out of nine). We considered the possibility for each breeder to exploit other donors, which would bring additional favorable segments not present yet in their respective elite lines or the donors that they have already used. To identify and prioritize such additional donors, we considered the concept of optimal haploid value thanks to the H criterion (Daetwyler et al. 2015; Allier et al. 2020b).

The posterior mean marker effects ($\widehat{{\varvec{\beta}}}$) from a BRR model calibrated using all data were used to define the haplotypic estimated breeding value matrix (HEBV), which contains the estimated genetic effects for each haplotype segment in each individual:

$$\mathbf{H}\mathbf{E}\mathbf{B}\mathbf{V}=\left[{\varvec{X}}\circ \left({1}_{{\varvec{n}}}\widehat{{\varvec{\beta}}}\boldsymbol{^{\prime}}\right)\right]{\varvec{Z}}$$

where ${\varvec{X}}$ is the $[n\times m]$-dimensional genotyping matrix coded in 0 or 2 of the recipient and donor lines, ${1}_{{\varvec{n}}}$ is a vector of $n$ ones, $\circ$ denotes the Hadamard product. ${\varvec{Z}}$ is a $\left[m \times {n}_{H}\right]$ matrix of 0 and 1, where ${n}_{H}$ is the total number of haplotype segments considered. $\forall j \in \left[1,m\right] \ \text{and} \ \forall h \in \left[1,{n}_{H}\right]$, ${Z}_{jh}=0$ if the marker $j$ is not in haplotype segment $h$ and ${Z}_{jh}=1$ if the marker $j$ is in haplotype segment $h$. We considered haplotype segments of 100 SNPs with a 20 SNP increment, as recommended in Allier et al. ( 2019a).

For each recipient, the H criterion of each candidate donor was defined as:

$${H}_{\text{candidate}}=\lambda \sum_{h=1}^{nH}\text{max}\left\{{\text{HEBV}}_{\text{candidate},h}, {\text{HEBV}}_{\text{recipient},h}, \underset{D \in \text{Initial} \ \text{donor}}{\text{max}}\{{\text{HEBV}}_{D,h}\}\right\}$$

where $\text{Initial donor}$ is the set of donors already crossed with the recipient line. $\lambda$ is a scaling parameter defined as the ratio between the increment size (20) and the haplotype segment size (100), which equal to 0.2 in our case. $H$ reflects the maximum doubled-haploid line performance expected after several generations of intercrossing and selection. Donor incorporation order was determined following the forward selection procedure presented by Allier et al. ( 2019a).

For comparison, we also ranked the candidate donors for each elite line according to their predicted UC values. These predictions were performed using the procedure presented above, with a BRR model calibrated with a TS of RD type and the VPM method.

## Results

### Within-family mean performance and additive genetic variances

**Fig. 2:**
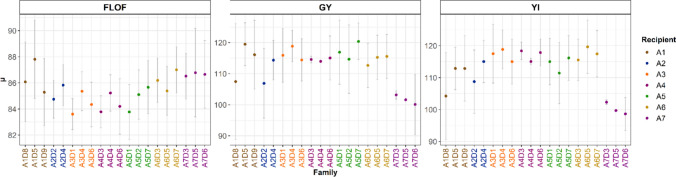
Visualization of the estimated mean performance ($\mu )$ and additive genetic variance of the observed crosses for female flowering (FLOM, in days), grain yield (GY, in 0.1t.ha^−1^) and yield index (YI, in 0.1t.ha^−1^). The estimations were performed with a GBLUP model undependably in each family. The vertical gray bars indicate ± two times the estimated additive genetic standard deviation for each family (colour figure online)

For FLOF, family mean ranged from 83.6 to 87.8 days after planting (Fig. 2 and Table S1). This range was lower for FLOM, from 83.3 to 86 days after planting. On average, the families derived from A3, A4 and A5 exhibited lower FLOF mean values than those derived from A1, A2, A6 and A7,-1.6 days on average. For FLOM, the means over the recipient lines were similar. Within-family additive genetic variances showed a mean value of 1.08 for FLOF. Those of FLOM were lower, with a mean value of 0.81. For both traits, the families produced from A1, A5 and A7 showed, in trends, higher additive genetic variances than other families. ASI had mean values ranging from − 0.82 to 1.71 days over the families. The families derived from A3, A4 and A5, which had the lower FLOF, had higher ASI mean values. The additive genetic variance for ASI was estimated close to 0 for some families, such as A2D2.

For GY, a large range of within-family mean values was observed, from 10.01 to 12.04 t.ha^−1^. The families derived from A7 underperformed others, with a mean value of 10.16 t.ha^−1^ compared to 11.43 t.ha^−1^ on average in other families. We also observed contrasted mean values between families from the same recipient line. For example, the GY mean performance of A1D8 was lower by 1 t.ha^−1^ than those of A1D5 and A1D9. In the same way, there was a difference of 0.75 t.ha^−1^ between the families A2D2 and A2D4 for GY. Conversely, the families derived from A4 had very close GY mean values (around 11.40 t.ha^−1^). These trends were also observed for YI.

The GY and YI mean additive genetic variances were 17.3 and 13.2, respectively. They showed high variation between families. For GY, this variance was almost zero in the A4D3, A4D4, A7D5 and A7D6 families, while it reached 86.2 in the A1D8 family. Families with comparable mean performances could present different additive genetic variance values, as illustrated by the families derived from A4, for which the three families had similar GY mean performances, but A4D6 had substantially higher variance.

For H2O, A1 and A5 progenies had slightly higher mean values. The different families had similar additive genetic variances, with a mean value of 0.64. Only A7D3 differed with a low variance value (0.07).

We observed an apparent influence of A4 on PH: The families derived from this recipient line had a mean value of 304.9 cm compared to 281.2 cm on average in other families. The PH additive genetic variances ranged from 3.28 to 82.44.

### Comparison of the within-family predictive abilities

The within-family PAs averaged over 100 repetitions differed between traits, with average values over families ranging from 0.18 (ASI) to 0.44 (H20). Mean PAs were higher for flowering time traits (FLOF: 0.41 and FLOM: 0.36) than for yield performance traits (GY: 0.26 and YI: 0.24). The standard deviation of the average PAs of families varied from 0.13 (FLOF) to 0.20 (GY), indicating significant variations between families (Fig. S1 and Table S2).

For all traits, higher PAs were reached for families derived from A1 (0.47 on average). Conversely, the A7 families had the weakest PAs, with an average value of 0.14 over traits. For other recipient lines, average value ranged from 0.23 to 0.38. The families derived from A2 and A4 showed higher mean PAs for H2O than for other traits. We also observed PA variations between families derived from the same recipient. For example, A1D9 had the highest GY mean PA (0.59), while this value was moderate (0.21) for the A1D5 family.

Weak (< 0.1) or negative PAs were observed for some trait-family combinations, especially for ASI, PH, GY and YI, for which five or six families were concerned. For GY and YI, most of these families were derived from the A4 and A7 recipient lines.

### Comparison of the training sets to predict the hybrid values of a family

**Fig. 3:**
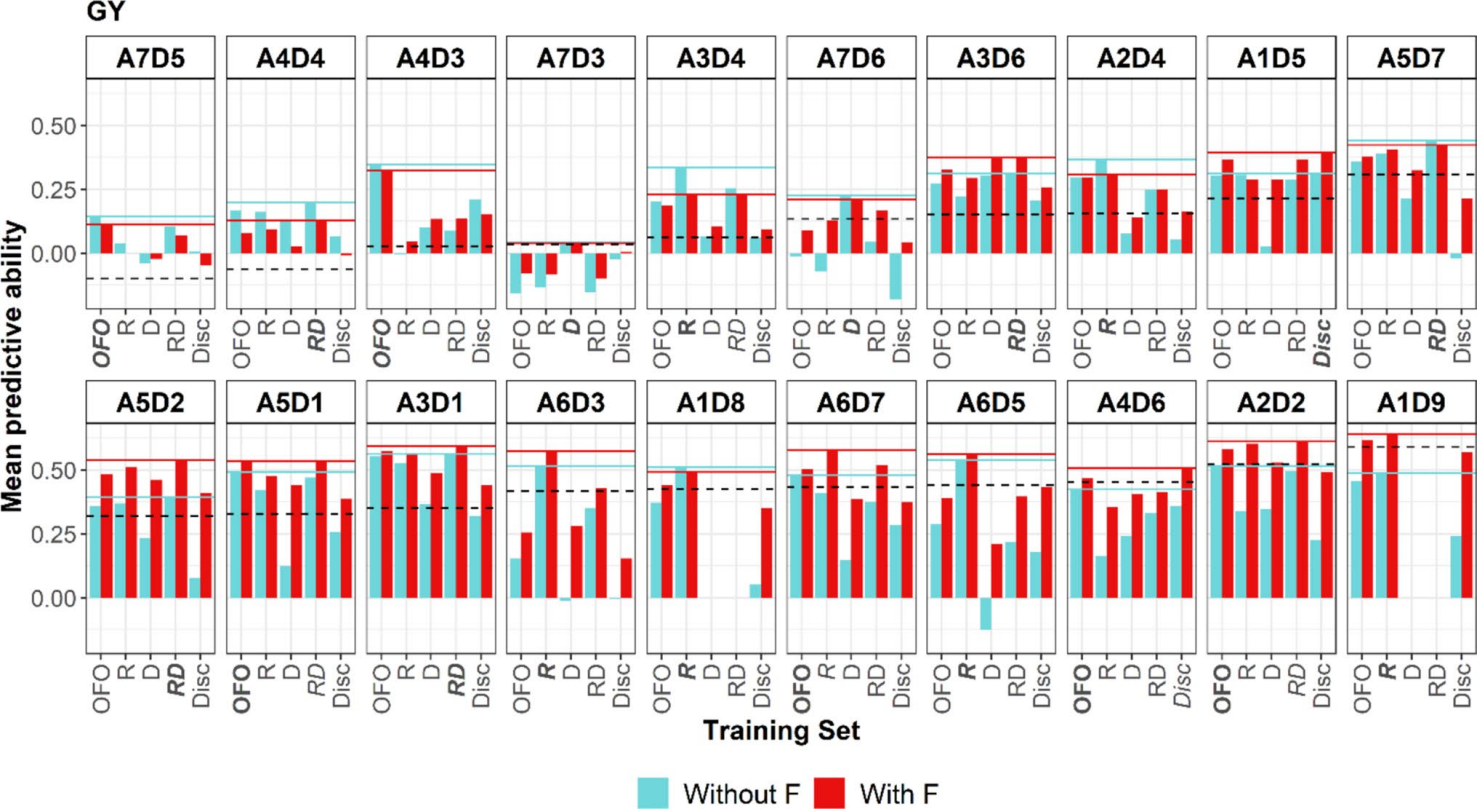
Mean predictive abilities (100 repetitions) obtained for predicting the grain yield values of individuals derived from each recipient x donor cross. For a cross and a training set type ($\text{F},\text{ OFO},\text{ R},\text{ D},\text{ RD or Disc})$, two-thirds of the individuals derived from the cross were sampled for use as a validation set. Their values were predicted using a GBLUP model calibrated with the individuals of the training set (*without F,* blue bars). For each cross, the training set with the highest predictive ability is bolded and the corresponding value is visualized with a blue line. Alternative training sets were also tested by adding the remaining third of the individuals derived from the cross (*with F,* red bars). For these alternative training sets, the one with the highest predictive ability is in italics and the corresponding value is visualized with a red line. The families are ranked by increasing values of within family predictive abilities (cross-validation with 100 repetitions) visualized by the gray dashed line (colour figure online)

For the vast majority of family–trait combinations, at least one of the inter-family TSs (OFO, R, D, RD or Disc) had a higher mean PA value than that observed for the within-family predictions (Table S2). For FLOF and FLOM and to some extent for GY and YI, we noticed a correlation between the PAs obtained with the inter-family TSs and those observed for the within-family TSs. With the OFO TS type, these correlations were 0.80 and 0.77 for FLOF and FLOM and 0.65 and 0.68 for GY and YI. For other traits, they ranged from 0.16 to 0.47. In particular, some families with low within-family PA were still poorly predicted by the different inter-family TSs (e.g., A7D5, A4D4 and A7D3 for GY in Fig. 3).

The highest inter-family mean PAs for FLOM were obtained with OFO or RD TS types for 17 out of 20 families. The PA averaged over all families was 0.43 for OFO and 0.39 for RD. The PAs obtained with OFO were similar to or slightly higher than those observed with RD. We noticed the largest differences for A3D6 and A7D3. In trends, calibrating the prediction model only with R or D TS types instead of RD led to a decrease in PA values, down to 0.26 (R) or 0.29 (D) on average. As expected, the Disc TS type showed the lowest PA values (0.18 on average). Similar trends were observed for FLOF and PH. For FLOF, the highest inter-family mean PAs were reached with OFO or RD for 19 out of 20 families with average values of 0.49 (OFO) and 0.45 (RD). OFO and RD were also the most performant TS types to predict ASI, with average PAs of 0.31 (OFO) and 0.28 (RD).

For GY, the highest PAs were found for 18 out of 20 families when OFO, R or RD TSs were used to calibrate the prediction model (Fig. 3). The same result was observed for YI for 17 families. For both traits, the average PA was identical for OFO and R (0.30 for GY and 0.31 for YI). RD was slightly behind, with an average PA of 0.28 for both traits. In trends, D and Disc TSs led to lower PA values (e.g., for GY, 0.13 for D and 0.14 for Disc on average). For GY, the TSs including only half-siblings of the predicted individuals (R, D or RD) were outperformed by OFO for only six families. For these families, Disc TSs had higher PAs. A similar trend was observed for YI. For H2O, we observed a clear advantage of using OFO compared to other TS types, with an average PA of 0.47 with this TS type.

Adding full siblings in the initial TSs (OFO + F, R + F, D + F, RD + F or Disc + F) had a beneficial impact with a global increase of the PA (Table S3). For example, we observed a mean increase of 0.09 in the PA values for the GY prediction (Fig. 3). This addition was unfavorable for families with low within-family PA values, such as A4D4 for GY. In this case, the PAs were intermediate between the initial and within-family PAs. The advantage of adding full siblings was more marked for TS types with the lowest PAs. As an illustration, for GY, we observed a mean gain of 0.11 and 0.14 for the D and Disc TSs. The PA increase was moderate for other TS types (OFO, R, RD), with a mean gain of 0.04.

### Comparison of the training sets to predict the UC value of a cross

**Fig. 4:**
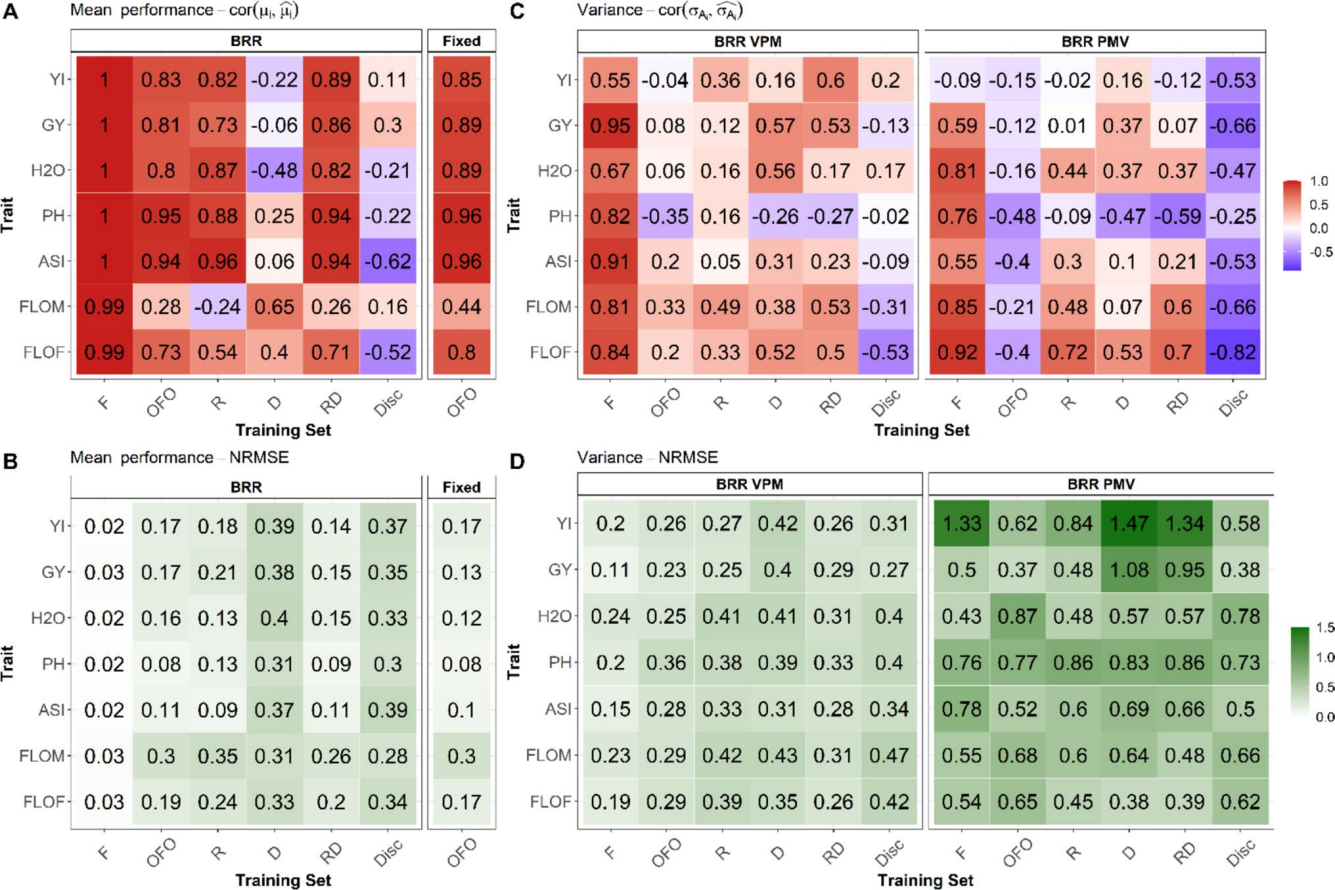
Predictive abilities and Normalized Root-Mean-Square Errors (NRMSEs) obtained for the prediction of the mean performance (**A**, **B**) and variance (**C**, **D**) of crosses. BRR: For a cross $i \in [\text{1,20}]$ and a training set type $j \in \{\text{F},\text{ OFO},\text{ R},\text{ D},\text{ RD},\text{ Disc}\}$, marker effects were estimated with a Bayesian Ridge Regression model calibrated with $j$ to predict the mean performance and the variance of $i$. Two variance prediction methods were compared: “variance of the posterior means” (VPM) and “posterior mean variance” (PMV). Fixed: For a cross $i \in [\text{1,20}]$, mean performances were predicted from the estimation of fixed parental effects with the training set type OFO. The predictive abilities for each trait and predicted parameter were the correlation between the observed (${x}_{\text{obs},i}$) and predicted (${x}_{\text{pred},i}$) values over twenty crosses. $\text{NRMSE}=\text{RMSE}/\left({x}_{\text{obs},\text{max}}- {x}_{\text{obs},\text{min}}\right)$ where ${x}_{\text{obs},\text{min}}$ and ${x}_{\text{obs},\text{max}}$ indicate the minimum and maximum values observed across families, respectively. $\text{RMSE}=\sqrt{\frac{1}{20}\sum_{i=1}^{20}{\left({x}_{\text{pred},i}-{x}_{\text{obs},i}\right)}^{2}}$

We predicted the cross means using marker effects estimated from a BRR model and the expected parental genome proportion in progeny. This method worked successfully when the individuals derived from the cross were used as calibration (F in Fig. 4 and Table S4). For calibrations involving progenies of other crosses, OFO, R and RD performed well for all traits except FLOM. Without considering FLOM, these TS types showed average correlations of 0.84 (OFO), 0.80 (R) and 0.86 (RD) between observed and predicted cross means, with NRMSE values close to 0.15. Conversely, D and Disc showed low or negative correlation values. For these TSs, we also observed a stronger NRMSE value (0.35). For FLOM, NRMSE values were higher than for other traits. The highest FLOM correlation was observed for D (0.65). For the different traits, predictions of cross-mean performances using estimations of fixed parental effects from $M6$ calibrated with OFO (“Fixed” in Fig. 4) led to higher PAs than the prediction with marker effects from the BRR model.

The prediction of the progeny variances was investigated by comparing (i) the “variance of posterior means” (VPM) and “posterior mean variance” (PMV) methods and (ii) diverse TS compositions (Fig. 4 and Table S5). PH cross variances were poorly predicted by both methods with the different inter-family TS types (OFO, R, D, RD and Disc), with negative correlations between observed and predicted cross variances and higher NRMSEs than other traits. Otherwise, OFO and RD associated with VPM were the most relevant to predict the cross variances with the lowest NRMSEs and the highest correlation values. In particular, these correlations ranged between 0.5 and 0.6 for the FLOF, FLOM, GY and YI variance predictions with RD.

For each cross and for each performance trait (GY and YI), we computed the observed UC value using the estimation of the mean and variance obtained with the model $M1$ (Fig. 2, Tables S1 and S6). Note that the mean contributed to 62,5% and 80,3% of the variation of UC for GY and YI, respectively. For the different TS, we predicted the UC by combining the predicted mean and variance values obtained with the model *M3* (Table 1). For both traits, the predictive abilities of the UC followed a similar trend to that of the mean (Fig. 4). High correlations between observed and predicted UCs, ranging from 0.83 to 0.91, were obtained using the VPM method with the OFO, R or RD TS types. For these TS types, the NRMSEs for the GY and YI UC predictions were similar to those obtained for the mean predictions. D and Disc led to high NRMSEs and weak correlations. The same trends were observed for PMV with lower correlation values between observed and predicted UC values and larger NRMSE values.

**Table 1:** Predictive abilities, RMSEs and NRMSEs obtained for the prediction of the usefulness criterion values of the grain yield (GY) and the yield index (YI)

		BRR VPM						BRR PMV					
		F	OFO	R	D	RD	Disc	F	OFO	R	D	RD	Disc
GY	${\mathbf{Cor(}}{\varvec{UC}}_{{\varvec{i}}} \user2{,}\widehat{{{\varvec{UC}}}}_{{\varvec{i}}} {\mathbf{)}}$	**0.96**	**0.83**	**0.91**	** − 0.23**	**0.88**	**0.29**	**0.86**	**0.81**	**0.88**	**0.07**	**0.82**	**0.27**
	RMSE	2.41	4.06	3.49	10.17	3.66	10.13	8.17	7.52	7.92	8.68	7.02	7.41
	NRMSE	0.09	0.16	0.14	0.39	0.14	0.39	0.32	0.29	0.31	0.34	0.27	0.29
YI	${\mathbf{Cor(}}{\varvec{UC}}_{{\varvec{i}}} \user2{,}\widehat{{{\varvec{UC}}}}_{{\varvec{i}}} {\mathbf{)}}$	**0.96**	**0.84**	**0.90**	** − 0.36**	**0.90**	**0.26**	**0.69**	**0.83**	**0.86**	** − 0.08**	**0.86**	**0.32**
	RMSE	2.64	4.18	3.48	10.83	3.58	10.10	9.78	7.64	8.78	9.50	7.71	7.59
	NRMSE	0.09	0.15	0.12	0.38	0.13	0.36	0.34	0.27	0.31	0.33	0.27	0.27

For a cross $i \in [\text{1,20}]$ and a training set type$j \in \{\text{F},\text{ OFO},\text{ R},\text{ D},\text{ RD},\text{Disc}\}$, a usefulness criterion value was computed using the mean performance ($\widehat{{\mu }_{ij}}$) and variance ($\widehat{{{\sigma }_{A}^{2}}_{ij}}$) predicted with a Bayesian Ridge Regression model calibrated with $j$: $\widehat{U{C}_{ij}}= \widehat{{\mu }_{ij}}+\text{i}* \widehat{{{\sigma }_{A}^{2}}_{ij}}$ where $\text{i}$ is the intensity selection (we took $\text{i}=2.07,$ corresponding to a selection rate of 5%). The variance values were predicted using two methods: “variance of the posterior means” (VPM) and “posterior mean variance” (PMV). For a given TS$j$, the predictive abilities for each trait were the correlation between the observed (${UC}_{ij}$) and predicted ($\widehat{U{C}_{ij}}$) UC values over twenty crosses. $\text{NRMSE}=\text{RMSE}/\left({UC}_{ij,\text{max}}- {UC}_{ij,\text{min}}\right)$ where ${UC}_{\text{obs},\text{min}}$ and ${UC}_{\text{obs},\text{max}}$ are the minimum and maximum usefulness criterion values observed across families, respectively, and $\text{ RMSE}=\sqrt{\frac{1}{20}\sum_{i=1}^{20}{\left(\widehat{U{C}_{ij}}-{UC}_{ij}\right)}^{2}}$.

### Incorporation of new donors

**Fig. 5:**
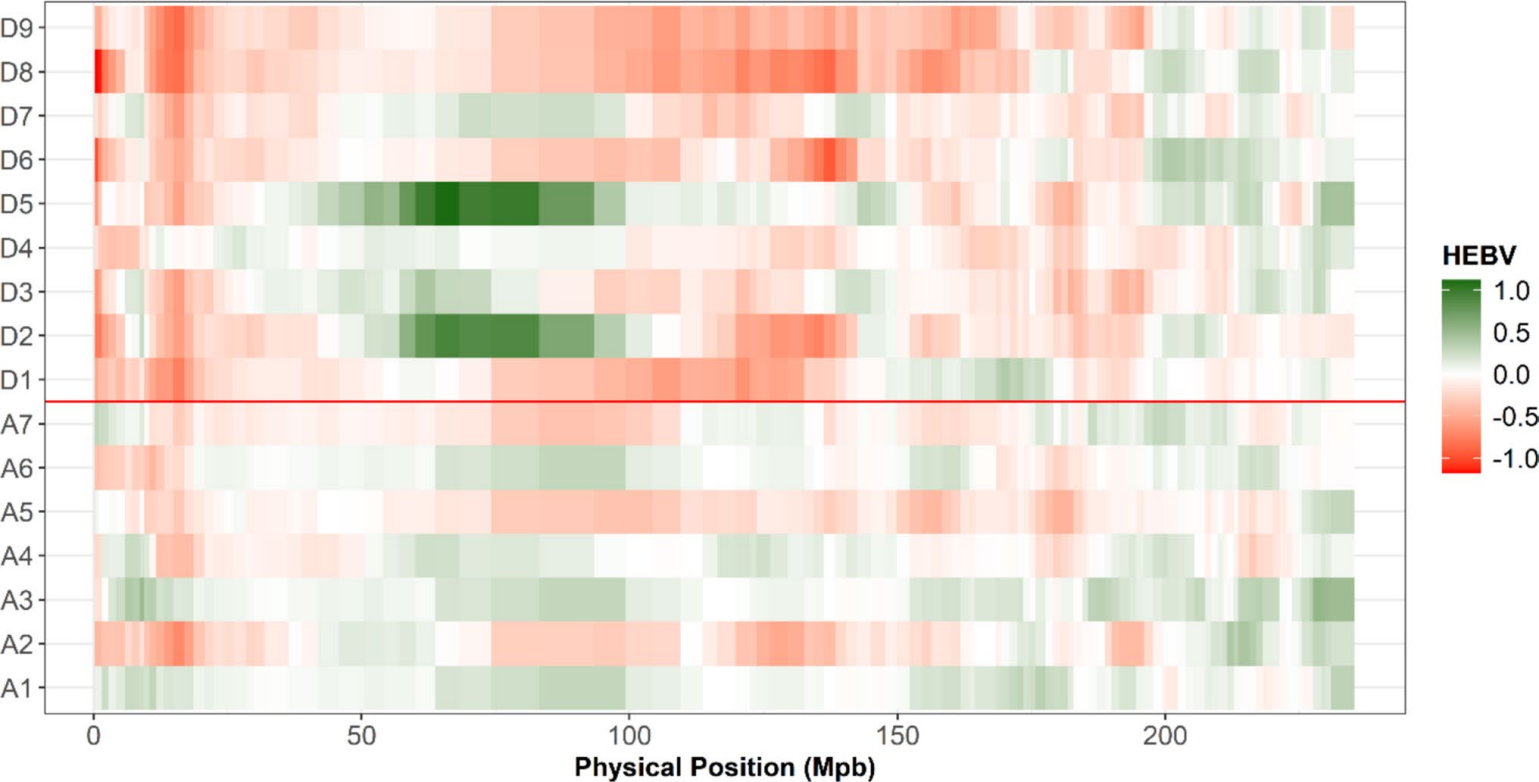
Visualization of the HEBVs with overlapping segments (100 SNPs with 20 SNP increment) for the grain yield along the chromosomes3. For each trait, the HEBVs were computed along the genome using marker effects estimated with a BRR model calibrated with all individuals of the dataset (twenty families). The x-axis represents the mean physical position of each haplotype segment in Mpb

The GY HEBV computation of donor and recipient lines revealed chromosomal regions where some donor lines had alleles superior to those of the recipient lines (Figs. 5, S2). As an illustration, we focused on two centromeric regions on the chromosomes 1 (112–150 Mpb) and 3 (60–93 Mpb) (Fig. S3). Only two recipients (A3 and A6) showed positive HEBVs for GY in the first region, while all donors had favorable haplotypes. For the second region, two donors (D2 and D5) stood out with high positive HEBVs for GY compared to other donors and the recipient lines (Fig. 5). We noticed that in the region on chromosome 1, the haplotypes with positive HEBVs for GY were also associated with later flowering (FLOF and FLOM), higher PH and higher H2O, while the region on chromosome 3 was specific to GY and YI (Figs. 5 and S3).

**Fig. 6:**
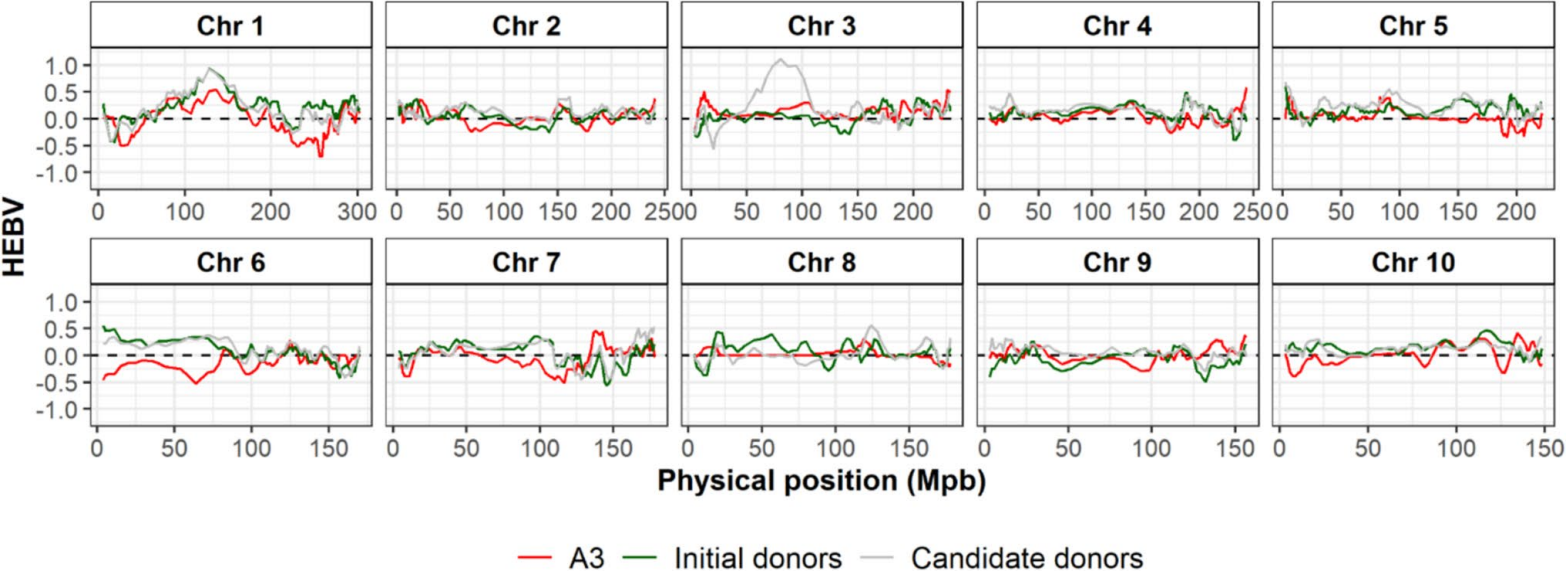
Visualization of the grain yield haploid estimated breeding values (HEBVs) with overlapping segments (100 SNPs with 20 SNP increment) along the genome considering the recipient A3 (red), donors already crossed with A3 (“Initial donors”, green) and candidate donors (gray). Initial donors: D6, D4 and D1. Candidate donors: D2, D3, D5, D7, D8 and D9. The HEBVs were computed using marker effects estimated with a BRR model calibrated with all individuals of the dataset (twenty crosses). The x-axis represents the mean physical position of each haplotype segment in Mpb (colour figure online)

**Fig. 7:**
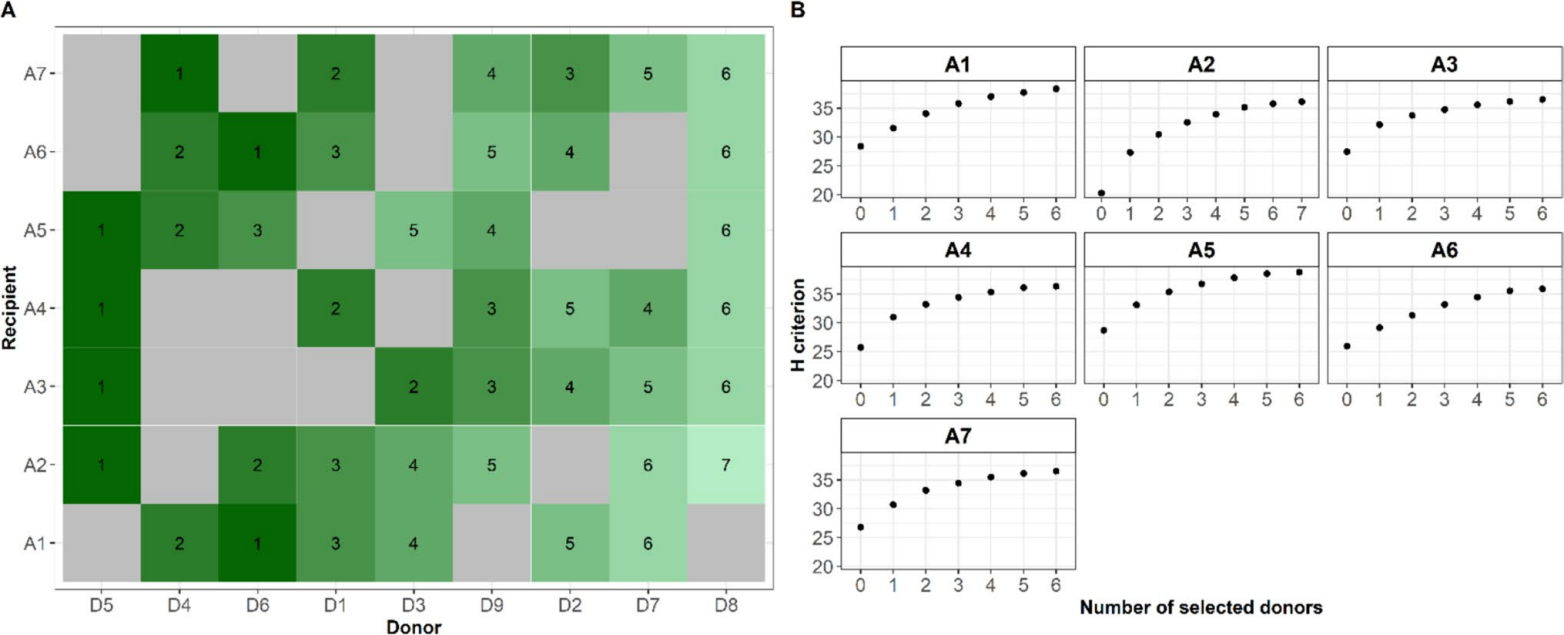
Result of the forward selection of donor lines using the H criterion. Each recipient line and donors already crossed with it (gray on A) were considered as the reference population to compute the H criterion values of remaining donors (green on A). The one with the highest H criterion value was selected as the next incorporated donor. The process was repeated until all donors were selected. The rank of selection (**A**) and the H criterion evolution (**B**) are reported (colour figure online)

For each recipient, visualizing GY HEBVs along the genome revealed regions of interest not incorporated yet in the crosses performed in our design (Fig. 6 for A3 and Fig. S4-9 for the other recipients). Based on the GY H criterion computation, we performed a forward selection among the candidate donors for each recipient to define which crosses should be privileged (Fig. 7). For recipients not crossed yet to D5, the H criterion always suggested integrating it first. For the other recipients, it prioritized a first cross with D4 or D6. On the contrary, D8 was always last to be selected. We observed an H criterion gain ranging from 10 to 25.7% after the virtual incorporation of the first donor (Fig. 7 and Table S7). Note that the highest value was obtained for A2, which had been crossed to two donors instead of three in our cross-design. For all recipient lines, the H criterion gain slowed down with the increase in the number of newly incorporated donors. A plateau occurred after the incorporation of three donors.

For each recipient, we also ranked the crosses not observed in our experimental design based on their predicted GY UC values. These values ranged from 10.43 (A7D8) to 12.70 t.ha^−1^ (A1D1), with an average intra-recipient standard deviation of 11.3 t.ha^−1^ (Table S7). For all recipient lines except A7, the donor line ranked first using the H criterion was not the one with the highest UC value (Tables S8 and S9). When the UC was used instead of the H criterion to rank donors to be incorporated, the average H gain due to the first incorporation dropped from 14.9 to 8.8%. We noticed that the loss was limited for A1 (from 10 to 8%) and A6 (from 10.9 to 8.2%). A2, A3, A4 and A5 required two or three donor incorporations with a UC-based ranking to reach the H values obtained after the first one with an H-based ranking.

## Discussion

### Variability of means and variances between families

The flint maize population studied in this paper was composed of individuals derived from BC1 crosses between elite lines provided by the different partners and diversity donors chosen for their (i) originality, (ii) hybrid performance, (iii) limited agronomic defaults and (iv) phenology compatible with the targeted environment. Sanchez et al. (2024) provided preliminary estimations of each family’s mean value and additive variance using a subset of trials (Blo19, Smh19 and Vil19). The evaluation of the whole set of 7 trials, including Rec21, Vily21, Sel21 and Ein21 trials conducted in 2021, confirmed limited within-family flowering time variances. It also corroborated the homogeneity of flowering time across families derived from the same recipient line (less than three days). This homogeneity of flowering time will facilitate the incorporation of these new materials in the breeding programs. For the production traits, our results confirmed the presence of large differences in genetic variance between families derived from a same recipient and different donors, and those derived from different recipients. These experimental results support the interest of the Usefulness Criterion (UC), which considers these variances, to detect the most interesting donor x elite crosses (Schnell and Utz 1976; Zhong and Jannink 2007).

### Heterogeneity of the GS efficiency to predict the progeny values of a donor x recipient cross

The within-family GS prediction led to low or moderate predictive abilities (PAs). FLOF, FLOM and H20, which showed higher heritability values compared to other traits (Table S1), had also higher PA values, as expected from GS theory (Hayes et al. 2009). For all traits, we observed large variations of PAs between families. Notably, the families derived from the A7 recipient had lower PAs than others. These lower performances are likely due to the smaller size of these families.

The results obtained for the inter-family predictions were encouraging, with an increase of PAs compared to the within-family prediction (OFO TS). This improvement must be partly due to the increase in the TS size, which is known to positively impact prediction efficiency (Daetwyler et al. 2008; Zhong et al. 2009). The comparison of different TSs (R, D, RD and Disc) confirmed the influence of the level of relationship between the individuals of the TS and those to predict. TSs composed with only half-siblings of the individuals of the VS (R, D or RD) reached similar PA values than OFO. The PAs dropped significantly if no individual closely related to the target family was present in the TS (Disc). In this case, using individuals from the targeted family to complete the TS had generally a beneficial impact on the PAs. These findings encourage implementing GS in a bridging population to predict the value of individuals derived from a cross between a donor line and an elite line using individuals derived from either of these lines as TS. When the parents have not already been involved as parents of the crosses used to calibrate the GS model, at least a partial phenotypic evaluation of progeny of the donor x elite cross is requested.

### Prediction of the usefulness criterion

The UC of a cross involves a linear combination of the expected mean and variance of progenies. Accurate predictions of both parameters are needed to efficiently identify promising crosses between diversity donors and elite recipient lines. Predicting mean values from genome-wide marker effects worked well with the OFO TS type. The correlation between observed and predicted mean values was lower for FLOM, and to some extent FLOF, than for other traits. This is likely due to the studied crosses being poorly contrasted for flowering time due to the selection of parents with the same precocity. Adeyemo and Bernardo (2019) conducted a similar analysis with maize crosses exhibiting a larger FLOF variability and showed that the mean values for this trait were successfully predicted using marker effect estimations.

To predict progeny variances based on Bayesian estimations of marker effects, we compared the “variance of posterior means” (VPM) and “posterior mean variance” (PMV) methods proposed by Lehermeier et al. (2017). PMV was expected to lead to unbiased variance predictions by using the posterior mean from all samples to consider the uncertainty of the marker effect estimations (Lehermeier et al. 2017). Our results went against this initial expectation since PMV led to predicted variances higher than the observed ones, with higher RMSEs than VPM. Having biased progeny variance predictions may lead to misjudging the variance contribution to the UC and misclassifying the crosses. The opposition between our findings and the theoretical expectation may be related to the presence of non-additive effects, not considered in the simulation study proposed by Lehermeier et al. (2017), which may interfere with the estimation of progeny variances or those of marker effects. We also investigated the prediction of progeny variance from marker effects backsolved from a GBLUP model (Table S5). The results were similar to those obtained with VPM.

Our results confirmed that using genome-wide marker effects is less efficient in predicting progeny variances than in predicting progeny mean values (Lian et al. 2015; Adeyemo and Bernardo 2019; Neyhart and Smith 2019). For the mean values, having individuals derived from the recipient line in the TS (R, RD or OFO) was a minimal prerequisite for effective predictions, while the D TSs performed poorly. This asymmetry between the R and D TSs is likely due to the mating cross (BC1), which led to a higher representation of the recipient alleles in progenies. Conversely to the mean, only segregating markers in progenies participate in the variance definition. Accurately predicting the variance relies on estimating the effects of the markers segregating between the donor and the recipient. To do this, using only the R TS revealed to be insufficient. Adding individuals derived from the donor line to the TS (RD) increased correlations between observed and predicted variances and decreased NRMSEs (with the VPM method). Adding less related individuals in the TS (OFO) had a slightly negative or neutral impact on mean predictions, while it was largely detrimental for variance predictions. This finding supports using training sets adapted to each cross to predict variances, as discussed in (Lian et al. 2015).

The pattern for UC predictions was similar to those observed for mean value, with a clear advantage to the OFO, R and RD TSs. As for variance predictions, using specific TS (R and RD) led to the highest correlation values between observed and predicted UCs. Note that the correlation values were higher than those for the mean values. This trend was also observed in Neyhart and Smith (2019) but not in other studies (Adeyemo and Bernardo 2019; Wolfe et al. 2021). It may be due to sampling, given the limited number of evaluated crosses.

### Criteria to select new donors to enrich a bridging population

We compared the interest of the UC and the H criterion to determine new promising donor x recipient crosses to complement those already evaluated. The H criterion focusses on the presence of favorable original alleles in potential donors absent from the elite materials, which is key to increase long-term genetic gain (Allier et al. 2020b). Using a forward selection procedure enables one to consider a potential donor’s originality in relation to the previously introduced donors. The H criterion allowed us to identify three donor lines (D5, D4 and D6) that best complement the studied elite lines for GY. Using the UC to select donors led to a decrease in the H values, consistent with the fact that this criterion ignored the previous crosses. On the other hand, contrary to the H criterion, the UC considers the performance value of the donor lines and a realistic occurrence of recombination events along the genome. Thus, its use is more likely to fill short-term genetic gain objectives. Short- and long-term objectives may be addressed thanks to a combination of both criteria. Allier et al. (2020b) proposed to use them sequentially by pre-selecting a set of donors for their global complementary to the elite pool based on the H criterion and defining the mating design between this set of donors and the elite lines to maximize the mean UC of crosses. The global pre-selection of donors for the whole set of elites may lead to miss some promising elite x donor combinations. To get around this constraint, we suggest to combine both criteria into a linear index giving a different weight to each. Preliminary tests with our dataset showed that candidate donor ranking differed according to the used weight (**Fig S10**). Simulating breeding programs conducted using this index may be valuable to quantify its impact on genetic gain with different weight values and find the optimal balance between both criteria.

With the H criterion, which necessitates the computation of HEBVs, it is also possible to refine donor introductions by targeting some specific chromosomic regions, particularly those with low diversity in elite material. The comparison of HEBVs of the donor and elite lines used as parents of our population highlighted two centromeric regions on chromosomes 1 and 3, for which donors carried favorable haplotypes absent from the elite lines for GY. Introgressing these regions in elite materials should be considered to improve their performance and reboost the local diversity. For the second one, we did not detect obvious negative pleiotropy with flowering time traits, which may facilitate its introgression. The interest of this genotypic region must be confirmed by performing genome-wide association study on a panel incorporating a larger number of non-elite and elite flint lines. The presence of the unfavorable haplotype in elite lines from different breeders may be due to the restricted number of lines used as parents of elite flint lines and a low recombination rate in this region (Messmer et al. 1992; Dubreuil and Charcosset 1999; Van Inghelandt et al. 2010).

### Guidelines for bridging population implementation supported by genomic selection

Implementing a bridging population requires (i) defining the initial donor x elite crosses, (ii) determining the bridging individuals to incorporate into the elite program and (iii) incorporating new donors to maintain diversity. For the initialization of the bridging population, we propose choosing the donors on their phenotypic values, which are more relevant than predictions using the elite population as TS (Sanchez et al. 2024). This evaluation may be coupled to H criterion computation to determine the complementarity of the donors with the elite materials and, to some extent, the number of donors to incorporate. The prediction of UC may also support the definition of the first donor x elite crosses, but further investigations are needed to determine the optimal procedure. We recommend having a balanced design between donor and elite lines.

Before introducing new donors, we propose considering the crosses not already done between parental lines of the bridging population and initiating those with the highest GS-predicted UCs. We recommend using specific TSs (R or RD types) to predict each UC cross, as these led to more accurate predictions than a global TS including all bridging individuals. The same TSs can be used to predict the progeny values of new crosses and then guide the incorporation of selected individuals into the elite population. A simpler alternative could be to perform predictions considering a single TS including all information available but at the cost of a slight reduction in predictive abilities. Bridging population implementation must be supervised by monitoring the elite diversity to guarantee the incorporation of original variation (Woolliams et al. 2015; Allier et al. 2020a). A decrease in elite diversity should engage new donor incorporations in the bridging population. We recommend partially phenotyping the progenies of newly incorporated donors to maintain accurate GS predictions. Our study tested prediction scenarios with one-third of the progeny that is phenotyped, but this proportion must be refined.

### Future work

Our work documented the impact of the TS composition on the estimation of the marker effects and the prediction of progeny variance. Variance prediction also depends on the recombination rate between markers. We assumed that the recombination rate was similar for all crosses and used the flint maize consensus genetic map delivered by (Giraud et al. 2014). However, the recombination rate might vary among crosses of the same species (Bauer et al. 2013), all the more that populations have diverged since a long time (Danguy Des Déserts et al. 2021). Our multi-parental population may be used to explore the interest of considering cross specific genetic maps to improve variance and UC predictions. In practice however, this would raise an issue for crosses for which no segregating population is available, calling for further research on the anticipation of the recombination based on parental information (Hsu et al. 2022).

Our study showed that predicting efficiently the progeny values or the UC of a new recipient x donor cross necessitates that the TS includes at least individuals derived from the recipient line, and that prediction efficiency increases when progeny of donor is also represented. Predicting the value of crosses of donors to new elite lines is key to benefit from the genetic gain that occurs in the elite program. This leads to consider to include in the TS elite individuals derived from crosses between the recipient line(s) already crossed to donors and other elite lines. Previous studies, conducted through simulations, have partially explored this question and indicated that including individuals from bridging and breeding (i.e., elite) populations in a shared TS can yield long-term benefits regarding genetic gain (Allier et al. 2020a; Sanchez et al. 2024). This suggests that future experiments close to ours should include bi-parental crosses between lines from elite population(s) in addition to donor x elite crosses. This would make it possible a global comparison of HEBVs of candidate donors with the whole range of variation currently segregating in the elite pool, which is what is needed to discover key donor haplotype segments.

## Supplementary Information

Below is the link to the electronic supplementary material.

Supplementary file1 (DOCX 6480 KB)

## Data Availability

The raw phenotypic data, their adjusted means per hybrid and donor genotypic data are available at: https://entrepot.recherche.data.gouv.fr/privateurl.xhtml?token=eaf2654a-723f-4bed-8c8f-d0e654f125a2
