# Supplementary material for: Genomic prediction-aided incorporation of genetic resources into elite breeding: lessons from a collaborative multiparental design in flint maize
Source: Theor Appl Genet. 2025 Oct 1;138(10):262. doi: 10.1007/s00122-025-05034-3 (PMC12488772; doi:10.1007/s00122-025-05034-3)
Supplement: Supplementary file 1 — Supplementary file1 (DOCX 6480 KB) [file 122_2025_5034_MOESM1_ESM.docx]

# Supplementary Material for:

**Genomic prediction aided incorporation of genetic resources into elite breeding: lessons from a collaborative multiparental design in flint maize.**

Dimitri Sanchez^1,2^, Sarah Ben Sadoun^1^, Tristan Mary-Huard^1,3^, Cyril Bauland^1^, Carine Palaffre^4^, Bernard Lagardère^4^, Delphine Madur^1^, Valérie Combes^1^, Stéphane Melkior^5^, Laurent Bettinger^2^, Alain Murigneux^6^, Laurence Moreau^1^, Alain Charcosset^1,*^

^1^ Université Paris-Saclay, INRAE, CNRS, AgroParisTech, Génétique Quantitative et Evolution – Le Moulon, Gif-sur-Yvette, 91190, France

^2^ LIDEA FRANCE, Avenue Gaston Phoebus, Lescar, 64230, France

^3^ Université Paris-Saclay, AgroParisTech, INRAE, UMR MIA-Paris Saclay, 91120, Palaiseau, France

^4^ UE 0394 SMH, INRAE, 2297 Route de l’INRA, Saint-Martin-de-Hinx, 40390, France

^5^ RAGT2n, Druelle, 12510, France

^6^ Limagrain Europe, 28 route d’Ennezat, Chappes, 63720, France

^*^Corresponding author: [alain.charcosset@inrae.fr](mailto:alain.charcosset@inrae.fr)

# Supplementary Material and Methods

## Trial structure and data control

In each trial, hybrids derived from the same recipient line were gathered in a sub-trial to minimize experimental errors in their comparison. In Blo19, Smh19 and Vil19, all sub-trials had the same dimension (216 plots). 27 hybrids of the A7 sub-trial showed germination issues in the 2019 trials and were discarded for the 2021 trials. In Rec21, Vily21 and Sel21, all sub-trials contained 216 plots except A7 (108 plots). In Ein21, each sub-trial contained 240 plots (A1-A6) or 120 plots (A7). Each sub-trial was divided into 12 blocks (6 blocks for A7 in2021 trials). A commercial hybrid (ADEVEY) and a reference hybrid (the cross between the recipient and tester for each sub-trial) were used as checks. These were repeated in each block. A subset of experimental hybrids was replicated within a given trial. On average, the number of repetitions per experimental hybrids varied across trials from 1.10 (Vily21) to 1.25 (Ein21) and an experimental hybrid was observed 7.2 times on average over the seven trials. Digger R package was used to define experimental plans to ensure a homogeneous repartition of checks and repeated experimental hybrids in each sub-trial (Coombes 2009).

Plot having a plant density inferior to the median density minus 15 plants were discarded. In Vil19, final plant density was not recorded and fresh grain weight was used as a proxy to filter aberrant plots (plots with a fresh grain weight below 7 kg were eliminated). On average, 6.5% of the plots were filtered out over the seven trials. Some sub-trials had a higher elimination rate because of a low germination rate (A7 sub-trials with 22% of plots over the seven trials) or a planting error (in Vily21, A2 sub-trial with 14% of plots). In 2021, we also observed a lower plant density in the A3 sub-trials, which led to discarding 20% of the plots.

## Spatial correction and adjusted means

For each trial, the raw phenotypic data were corrected for spatial effects estimated using autoregressive AR1xAR1 models. For each trait, the following model was fitted:

$$Y_{sjkhm}^{R}= \mu+ \eta_{s}+ \rho_{j}+\theta_{k}+ A_{h} +P_{h}+S_{sjkhm}+ E_{sjkhm} (M1)$$

$$\boldsymbol{A}\sim N\left( 0,{\boldsymbol{K}\sigma}_{A}^{2} \right) ,\boldsymbol{P}\sim N\left( 0,{\boldsymbol{I}\sigma}_{P}^{2} \right) iid, \boldsymbol{S}\sim N\left( 0,{\boldsymbol{\Sigma}_{\boldsymbol{c}}\left( \omega_{c} \right)\otimes\boldsymbol{\Sigma}_{\boldsymbol{r}}\left( \omega_{r} \right)\sigma}_{S}^{2} \right), \boldsymbol{E}\sim N\left( 0,{I\sigma}_{E}^{2} \right) ind$$

$$\boldsymbol{A}\perp\boldsymbol{P}\perp\boldsymbol{S}$$

where $Y_{sjkhm}^{R}$ is the raw performance of the repetition $m$of the hybrid $h$ belonging to the family $k$, derived from the recipient line $j$ and located in the sub-trial $s$. $\mu$ is the intercept, $\eta_{s}$ is the fixed effect of the sub-trial $s$, $\rho_{j}$ is the fixed effect of the recipient line $j$ and $\theta_{k}$ is the fixed effect of the family $k$. Note that one extra level was added to $\rho$ and $\theta$ to deal with the check hybrids (ADEVEY and the referent hybrids). For $\rho$, this level corresponded to ADEVEY. For $\theta$, it corresponded to ADEVEY and referent hybrids. $A_{h}$ is a random additive genetic effect, $P_{h}$ is a genetic permanent effect (modeling the genetic effect not captured by $A_{h}$). $S_{sjkhm}$ is a random spatial field effect and $E_{sjkhm}$ is the error term. $\boldsymbol{K}$ is a additive marker-based kinship matrix (see **Estimation of marker-based kinship matrices section** in the main text). $\boldsymbol{\Sigma}_{\boldsymbol{c}}$ and $\boldsymbol{\Sigma}_{\boldsymbol{r}}$ are the AR1 covariance matrices associated to rows and columns, respectively, with $\omega_{c}$ and $\omega_{r}$the auto-correlation parameters (Gilmour et al. 1997; Cullis et al. 1998). $\otimes$ refers to the Kronecker product. This model was compared to two sub-models differing by the inclusion of only one of two genetic effects ($A_{h}$ or $P_{h}$). These models were fitted using the ASREML R package (Butler et al. 2017). The model with the lowest Akaike information criterion (AIC) value was used for each trait to compute the corrected field plot values by subtracting spatial BLUPs (predicted from $S_{sjkhm}$) from the raw values. These corrected values were noted $Y^{C}$.

For each trait, adjusted means were computed over trials using a model considering a fixed hybrid effect:

$$Y_{lhm}^{C}= \mu+ \alpha_{l}+\beta_{h}+E_{lhm} \boldsymbol{E}\sim N\left( 0,{\boldsymbol{I}\sigma}_{E}^{2} \right) ind (M2)$$

$Y_{lhm}^{C}$ is the spatial corrected value of the repetition $m$ of the hybrid $h$in the trial $l$. $\mu$ is the intercept, $\alpha_{l}$ is the fixed effect of the trial $l$ and $\beta_{h}$ is the fixed genetic effect of the hybrid $h$. $E_{lhm}$ is the error term. All parameter estimations and genomic predictions were performed on the adjusted means of the hybrids thus obtained, noted $Y$ in the main text.

## Bibliography

Butler DG, Cullis BR, Gilmour AR, et al (2017) ASReml-R Reference Manual Version 4. VSN International Ltd, Hemel Hempstead, HP1 1ES, UK.

Coombes NE (2009) DiGGer, a spatial design program. Biometric Bulletin NSW Department of Primary Industries, Orange, NSW

Cullis B, Gogel B, Verbyla A, Thompson R (1998) Spatial Analysis of Multi-Environment Early Generation Variety Trials. Biometrics 54:1–18. https://doi.org/10.2307/2533991

Gilmour AR, Cullis BR, Verbyla AP (1997) Accounting for Natural and Extraneous Variation in the Analysis of Field Experiments. Journal of Agricultural, Biological, and Environmental Statistics 2:269–293. https://doi.org/10.2307/1400446

#
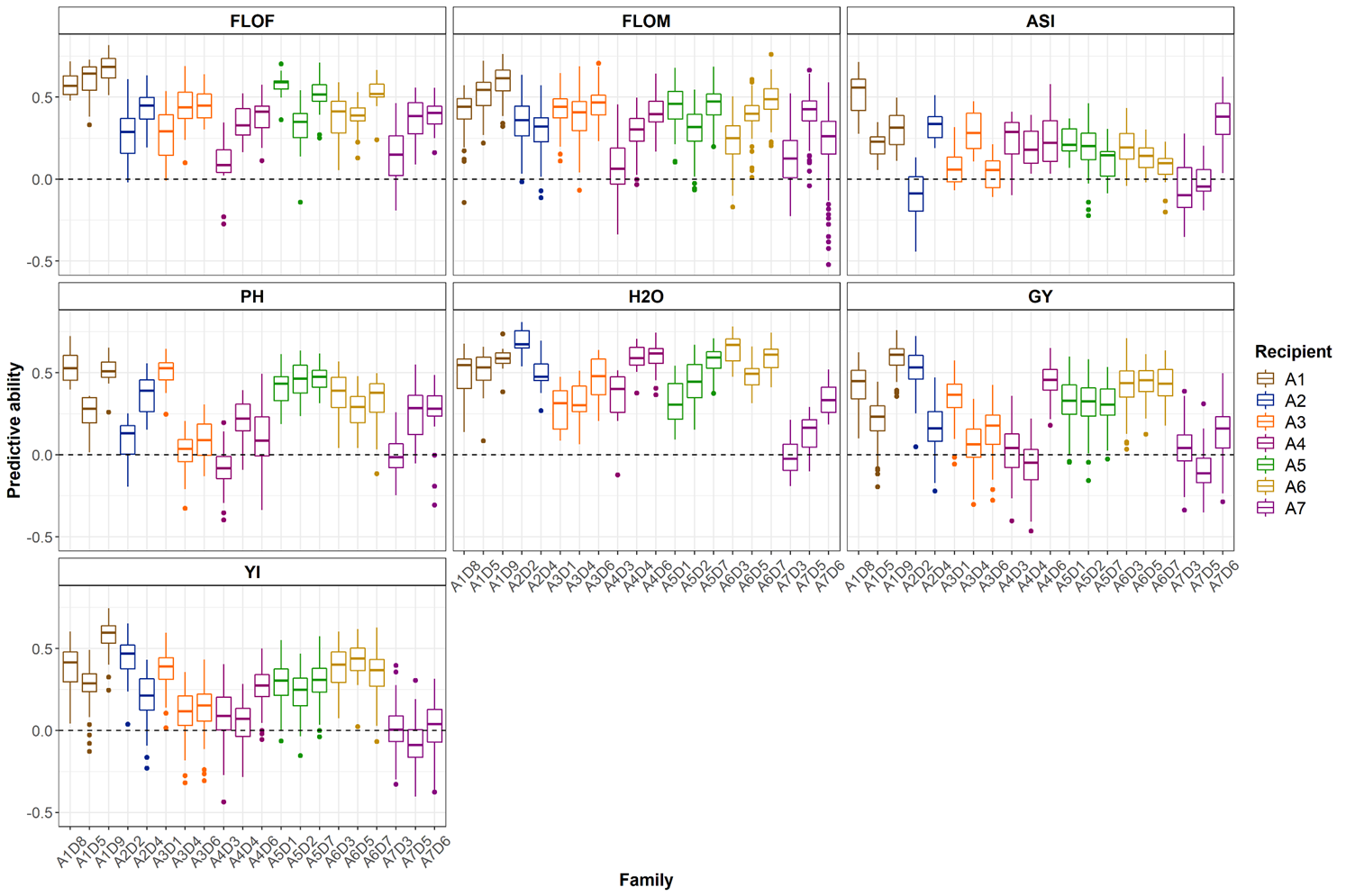
Supplemental figures

Fig. S1 Predictive abilities obtained for the within-family prediction of the performance values. For each family, one-third of the individuals of the family (derived from a recipient x donor cross) was sampled to calibrate a GBLUP model. This model was used to predict the remaining individuals’ values (100 repetitions).


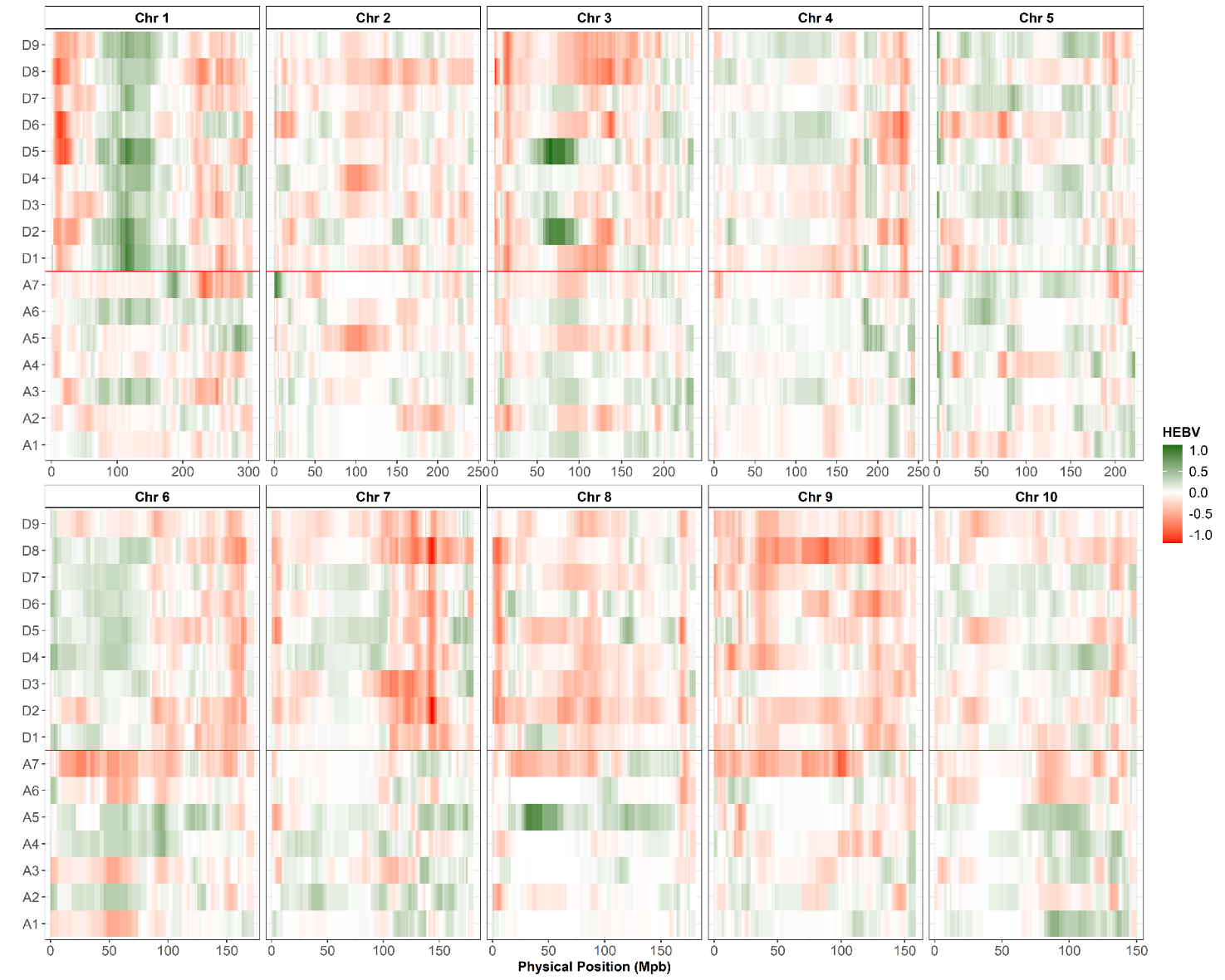
Fig. S2 Visualization of the grain yield HEBVs with overlapping segments (100 SNPs with 20 SNP increment) along the genome. The HEBVs were computed using marker effects estimated with a BRR model calibrated with all individuals of the data set (twenty crosses). The x-axis represents the mean physical position of each haplotype segment in Mpb.


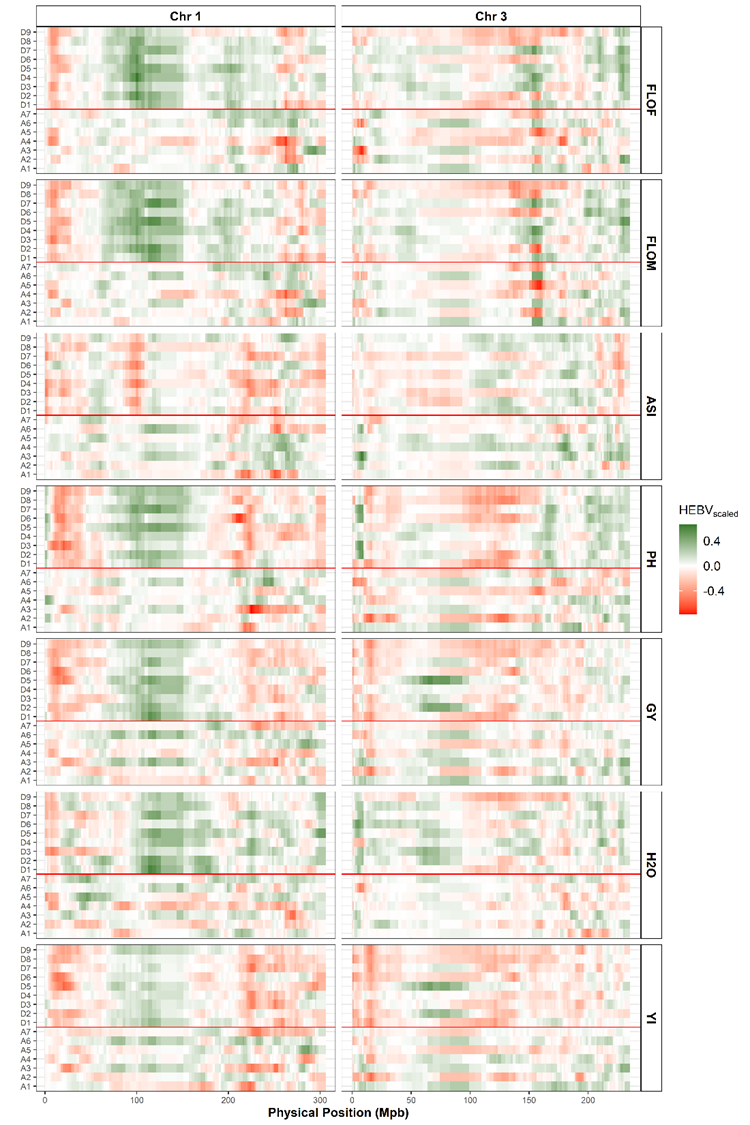
Fig. S3 Visualization of the HEBVs with overlapping segments (100 SNPs with 20 SNP increment) for the different traits along the chromosomes 1 and 3. For each trait, the HEBVs were computed along the genome using marker effects estimated with a BRR model calibrated with all individuals of the data set (twenty crosses). Only chromosomes 1 and 3 are displayed. HEBVs were scaled to facilitate the comparison between traits. For the individual $\boldsymbol{i}$, the trait $\boldsymbol{j}$ and the haplotype$\boldsymbol{k}$, $\boldsymbol{HEB}\boldsymbol{V}_{\boldsymbol{ijk, scaled}}\boldsymbol{=}\boldsymbol{100*HEBV}_{\boldsymbol{ijk}}\boldsymbol{/}\sum_{\boldsymbol{h=1}}^{\boldsymbol{H}} \left| \boldsymbol{HEB}\boldsymbol{V}_{\boldsymbol{ijh}} \right|$ where $\boldsymbol{H}$ is the number of haplotypes. The x-axis represents the mean physical position of each haplotype segment in Mpb.


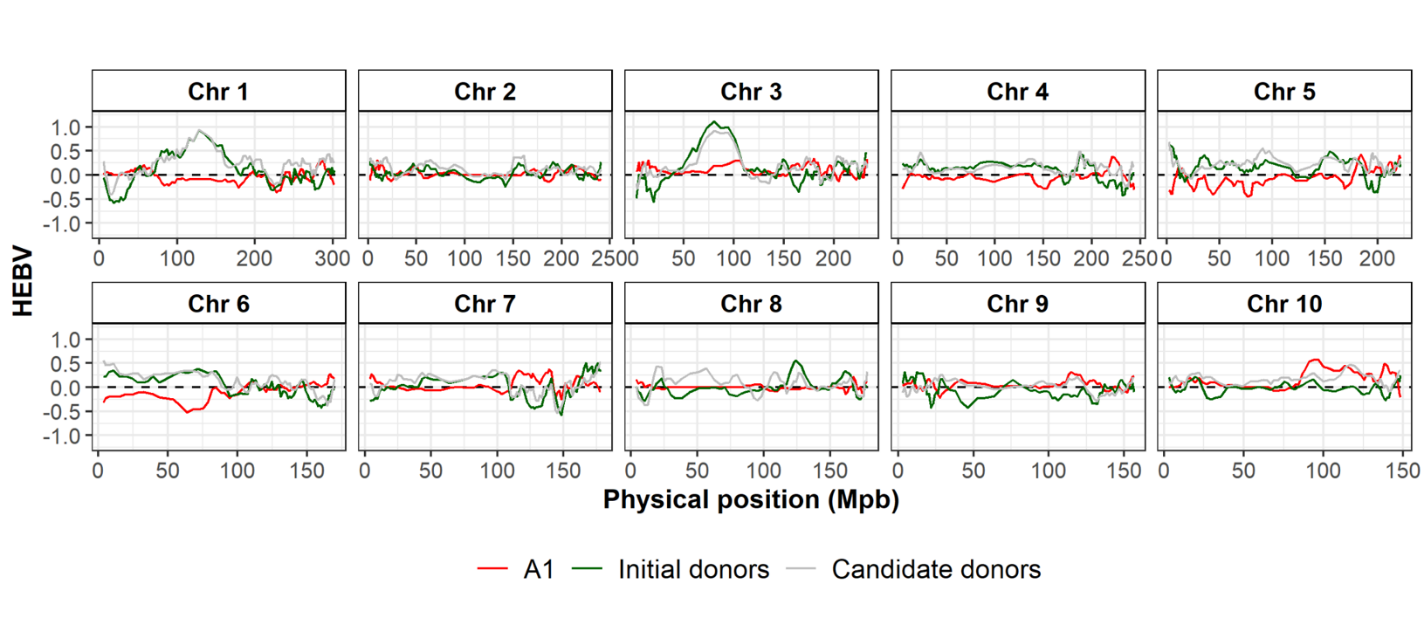
Fig. S4 Visualization of the grain yield haploid estimated breeding values (HEBVs) with overlapping segments (100 SNPs with 20 SNP increment) along the genome considering the recipient A1 (red), donors already crossed with A1 (“Initial donors”, green) and candidate donors (grey). Initial donors: D7, D8 and D9. Candidate donors: D1, D2, D3, D4, D5 and D6. The HEBVs were computed using marker effects estimated with a BRR model calibrated with all individuals of the data set (twenty crosses). The x-axis represents the mean physical position of each haplotype segment in Mpb.


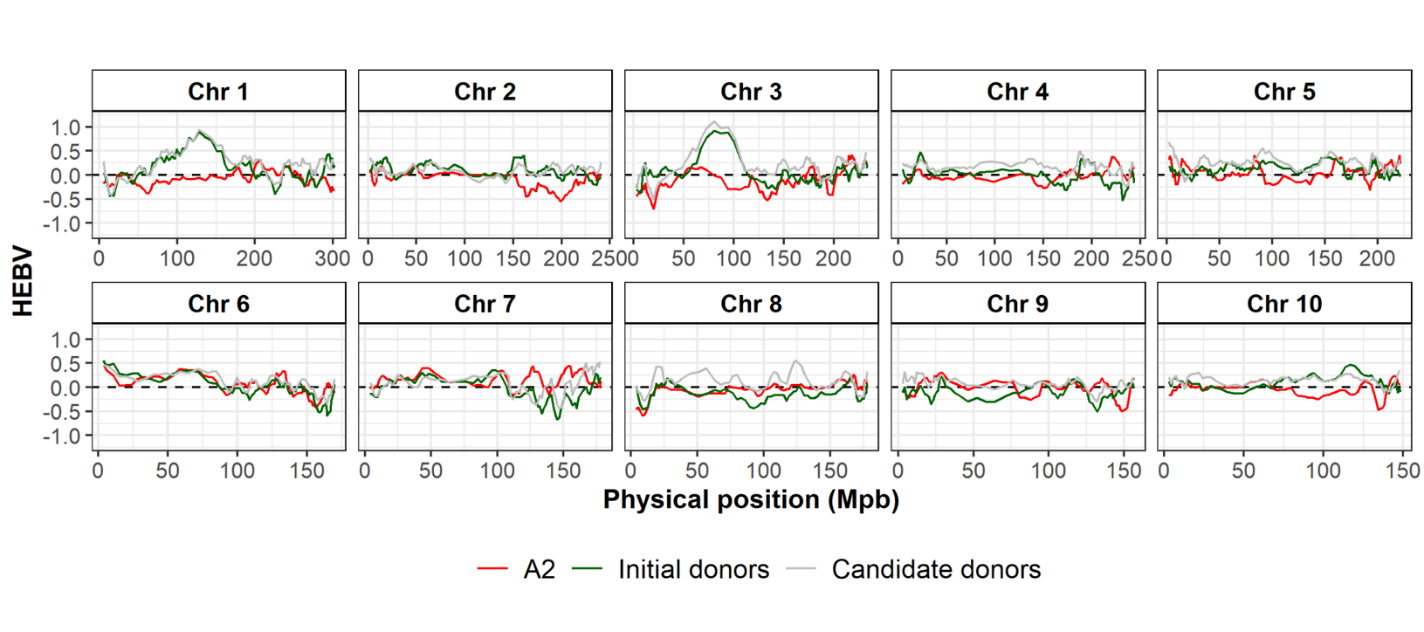


Fig. S5 Visualization of the grain yield haploid estimated breeding values (HEBVs) with overlapping segments (100 SNPs with 20 SNP increment) along the genome considering the recipient A2 (red), donors already crossed with A2 (“Initial donors”, green) and candidate donors (grey). Initial donors: D2 and D4. Candidate donors: D1, D3, D5, D6, D7, D8 and D9. The HEBVs were computed using marker effects estimated with a BRR model calibrated with all individuals of the data set (twenty crosses). The x-axis represents the mean physical position of each haplotype segment in Mpb.


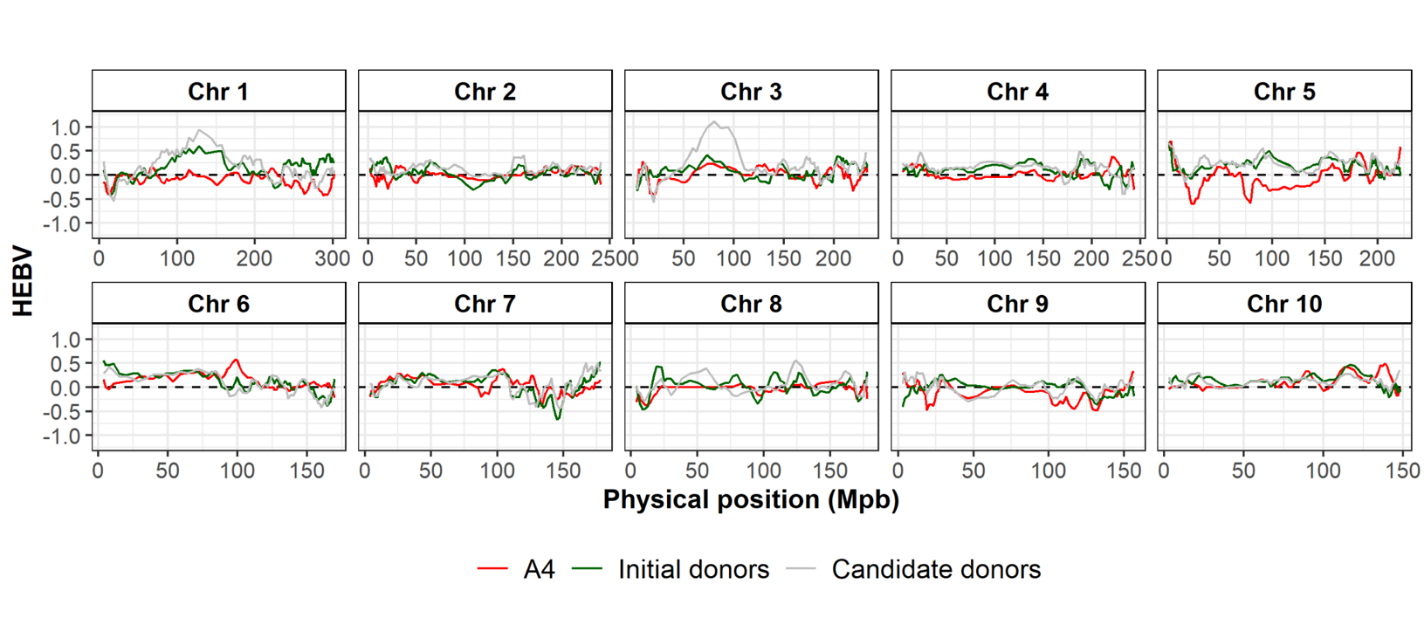
Fig. S6. Visualization of the grain yield haploid estimated breeding values (HEBVs) with overlapping segments (100 SNPs with 20 SNP increment) along the genome considering the recipient A4 (red), donors already crossed with A4 (“Initial donors”, green) and candidate donors (grey). Initial donors: D3, D4 and D6. Candidate donors: D1, D2, D5, D7, D8 and D9. The HEBVs were computed using marker effects estimated with a BRR model calibrated with all individuals of the data set (twenty crosses). The x-axis represents the mean physical position of each haplotype segment in Mpb.


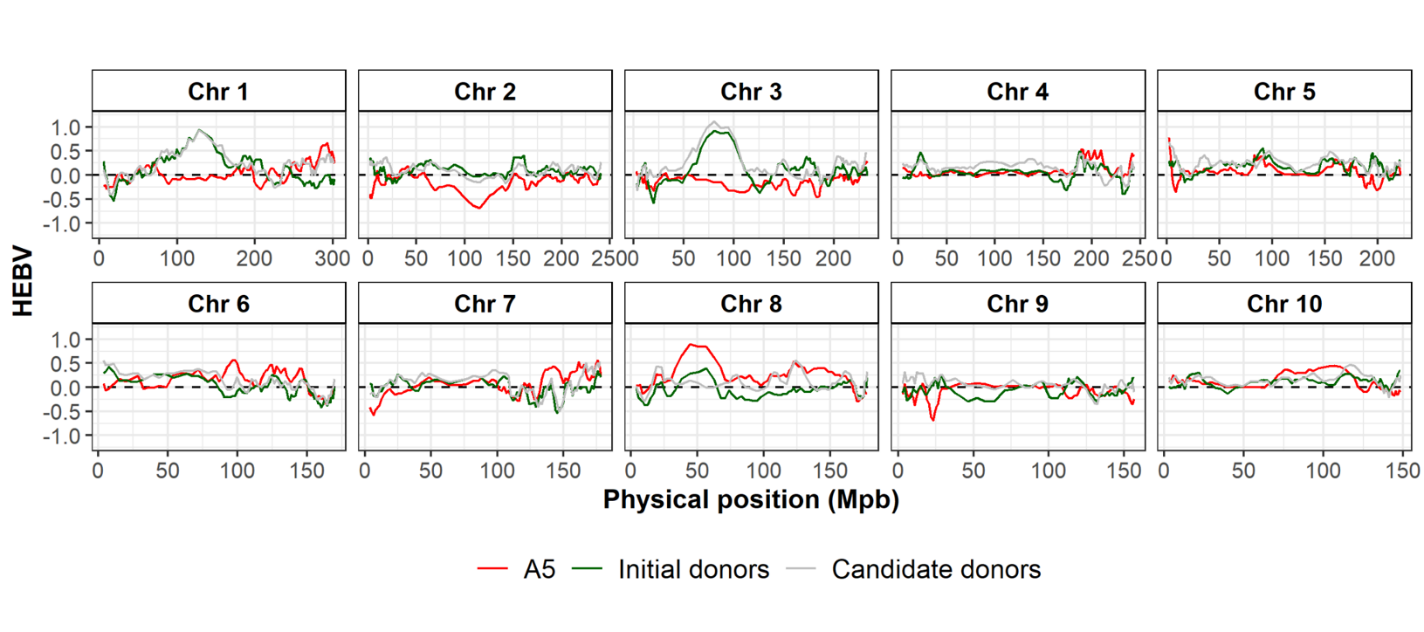


Fig. S7. Visualization of the grain yield haploid estimated breeding values (HEBVs) with overlapping segments (100 SNPs with 20 SNP increment) along the genome considering the recipient A5 (red), donors already crossed with A5 (“Initial donors”, green) and candidate donors (grey). Initial donors: D1, D2 and D7. Candidate donors: D3, D4, D5, D6, D8 and D9. The HEBVs were computed using marker effects estimated with a BRR model calibrated with all individuals of the data set (twenty crosses). The x-axis represents the mean physical position of each haplotype segment in Mpb.


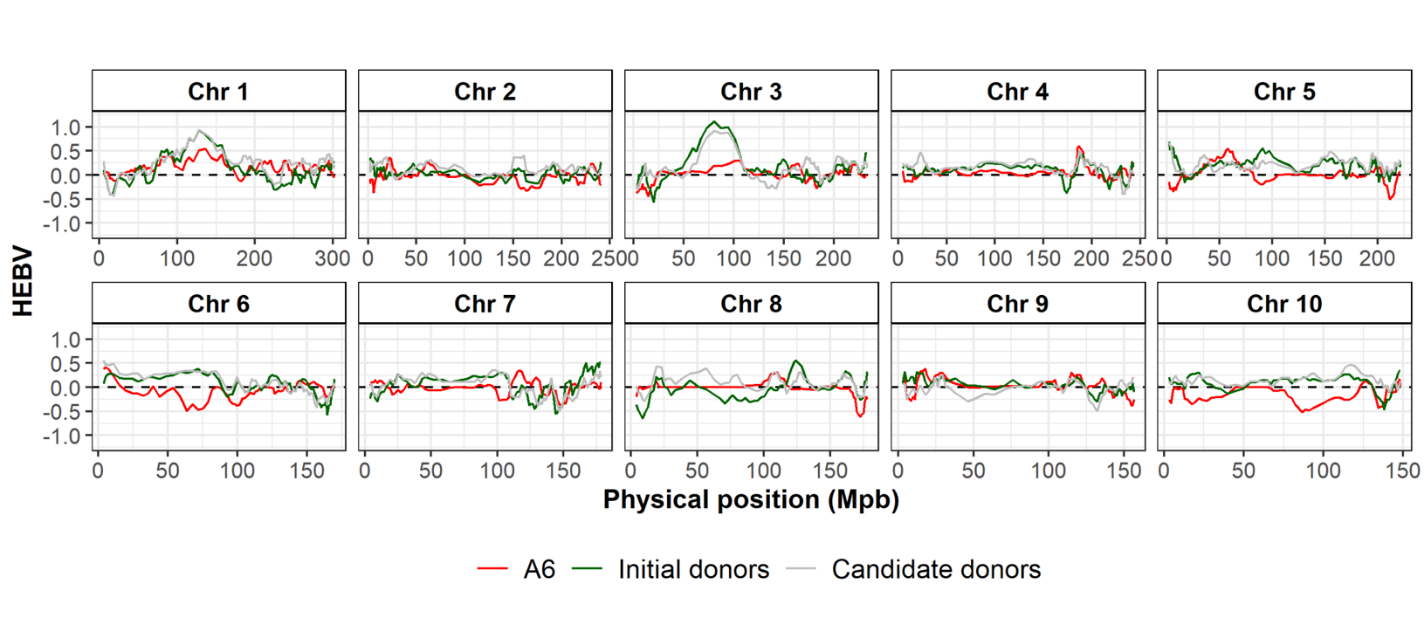
Fig. S8 Visualization of the grain yield haploid estimated breeding values (HEBVs) with overlapping segments (100 SNPs with 20 SNP increment) along the genome considering the recipient A6 (red), donors already crossed with A6 (“Initial donors”, green) and candidate donors (grey). Initial donors: D3, D5 and D7. Candidate donors: D1, D2, D4, D6, D8 and D9. The HEBVs were computed using marker effects estimated with a BRR model calibrated with all individuals of the data set (twenty crosses). The x-axis represents the mean physical position of each haplotype segment in Mpb.


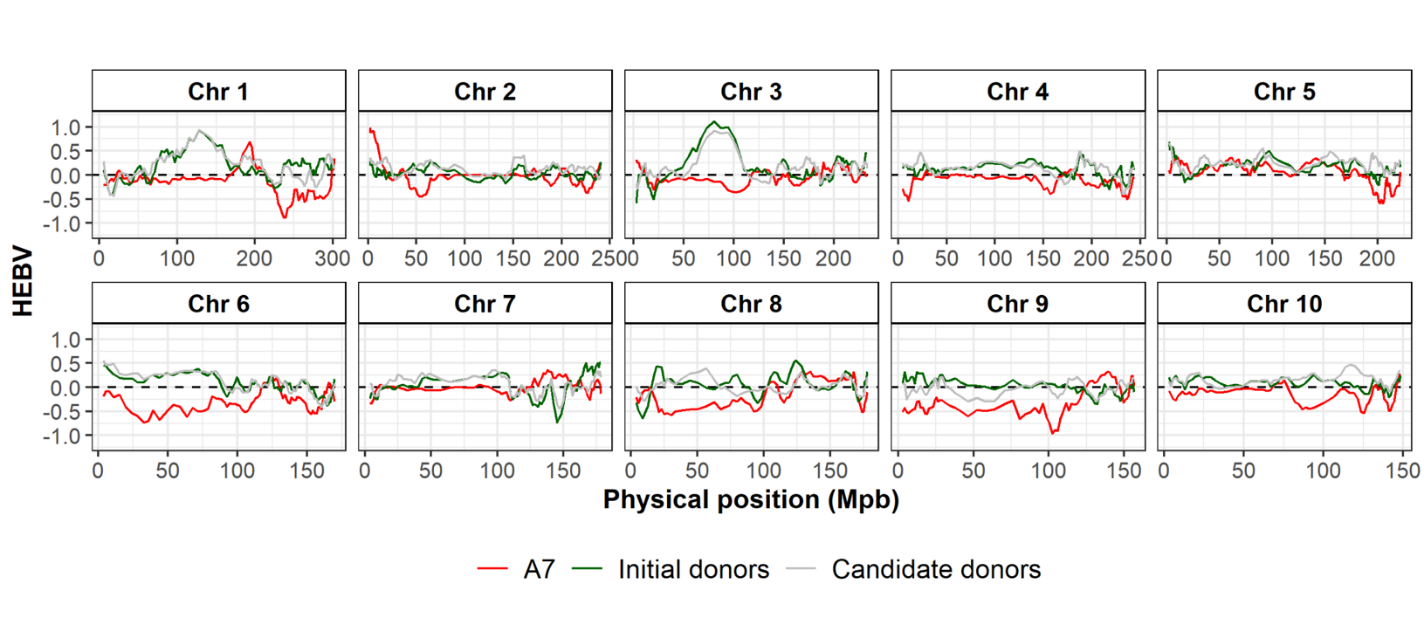


Fig. S9 Visualization of the grain yield haploid estimated breeding values (HEBVs) with overlapping segments (100 SNPs with 20 SNP increment) along the genome considering the recipient A7 (red), donors already crossed with A7 (“Initial donors”, green) and candidate donors (grey). Initial donors: D3, D5 and D6. Candidate donors: D1, D2, D4, D7, D8 and D9. The HEBVs were computed using marker effects estimated with a BRR model calibrated with all individuals of the data set (twenty crosses). The x-axis represents the mean physical position of each haplotype segment in Mpb.


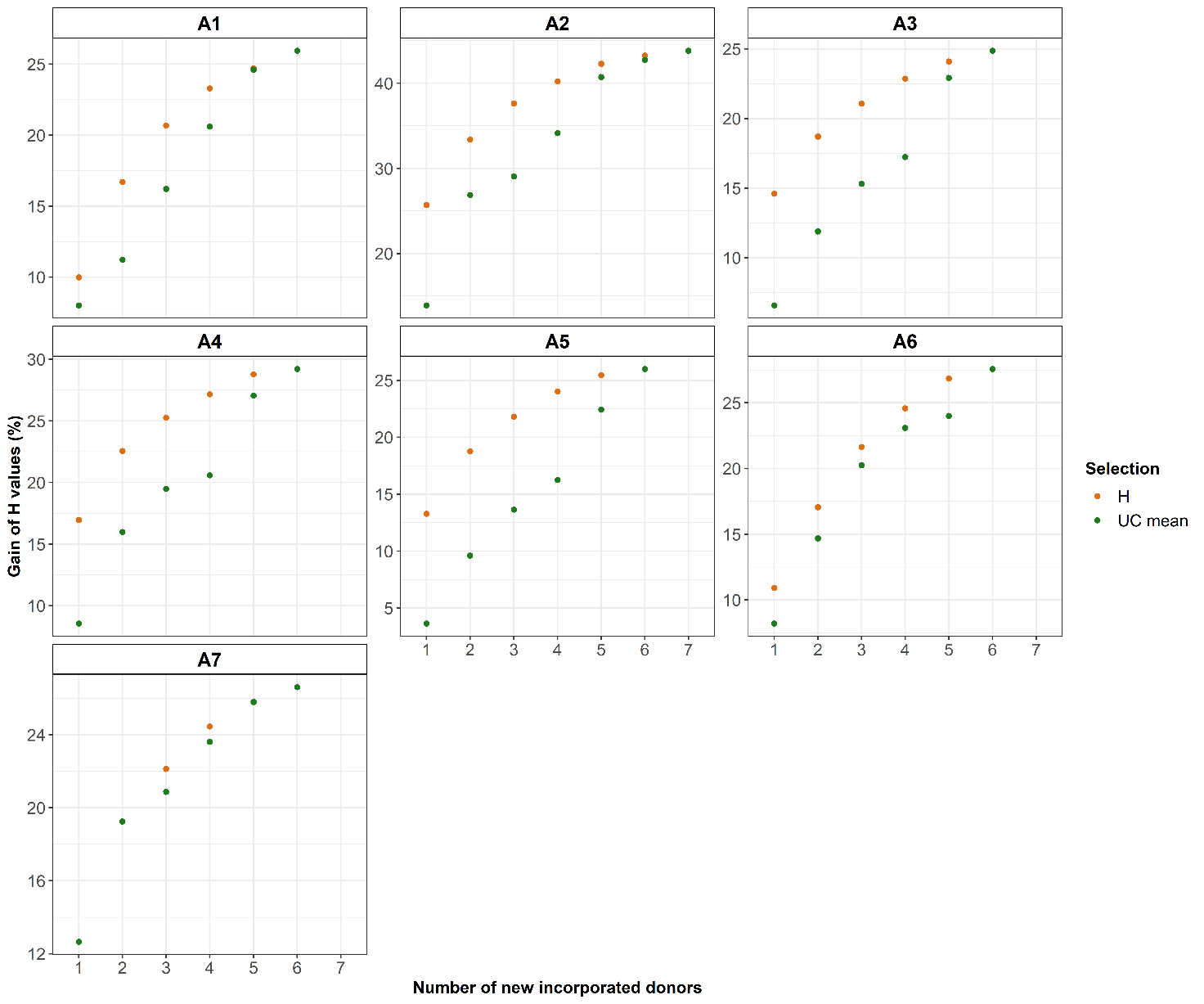


Fig. S10 Gain of the H criterion for each recipient line during a forward selection of new donor lines based on the maximization of a weighted score between this criterion and the mean usefulness criterion of the incorporated donors. For the selection of the $\boldsymbol{i}^{\boldsymbol{t}\boldsymbol{h}}$ new donor, a score $\boldsymbol{S}_{\boldsymbol{i}\boldsymbol{,}\boldsymbol{j}}$ is computed for each candidate donor $\boldsymbol{j}$as follows: $\boldsymbol{S}_{\boldsymbol{i}\boldsymbol{,}\boldsymbol{j}}\boldsymbol{=}\boldsymbol{\alpha}\boldsymbol{*}\frac{\bar{\boldsymbol{U}\boldsymbol{C}_{\boldsymbol{i}\boldsymbol{,}\boldsymbol{j}}}\boldsymbol{-}\bar{\boldsymbol{U}\boldsymbol{C}_{\boldsymbol{i}\boldsymbol{,}\boldsymbol{min}}}}{\bar{\boldsymbol{U}\boldsymbol{C}_{\boldsymbol{i}\boldsymbol{,}\boldsymbol{max}}}\boldsymbol{-}\bar{\boldsymbol{U}\boldsymbol{C}_{\boldsymbol{i}\boldsymbol{,}\boldsymbol{min}}}}$ + (1- $\boldsymbol{\alpha}$)*$\frac{\boldsymbol{H}_{\boldsymbol{i}\boldsymbol{,}\boldsymbol{j}}\boldsymbol{-}\boldsymbol{H}_{\boldsymbol{i}\boldsymbol{,}\boldsymbol{max}}}{\boldsymbol{H}_{\boldsymbol{i}\boldsymbol{,}\boldsymbol{max}}\boldsymbol{-}\boldsymbol{H}_{\boldsymbol{i}\boldsymbol{,}\boldsymbol{min}}}$ where $\bar{\boldsymbol{U}\boldsymbol{C}_{\boldsymbol{i}\boldsymbol{,}\boldsymbol{j}}}$ is the mean predicted UC value considering the previous incorporated donors and the candidate donor $\boldsymbol{j}$ (if $\boldsymbol{i}\boldsymbol{=}$1, $\bar{\boldsymbol{U}\boldsymbol{C}_{\boldsymbol{i}\boldsymbol{,}\boldsymbol{j}}}\mathbf{=}\mathbf{UC}_{\mathbf{j}}$), $\boldsymbol{H}_{\boldsymbol{i}\boldsymbol{,}\boldsymbol{j}}$ is the value of the H criterion after the incorporation of the candidate donor $\boldsymbol{j}$, $\bar{\boldsymbol{U}\boldsymbol{C}_{\boldsymbol{i}\boldsymbol{,}\boldsymbol{min}}}$ is the minimal value of $\bar{\boldsymbol{U}\boldsymbol{C}_{\boldsymbol{i}\boldsymbol{,}\boldsymbol{j}}}$, $\bar{\boldsymbol{U}\boldsymbol{C}_{\boldsymbol{i}\boldsymbol{,}\boldsymbol{max}}}$ is the maximal value of $\bar{\boldsymbol{U}\boldsymbol{C}_{\boldsymbol{i}\boldsymbol{,}\boldsymbol{j}}}$, $\boldsymbol{H}_{\boldsymbol{i}\boldsymbol{,}\boldsymbol{min}}$is the minimal value of $\boldsymbol{H}_{\boldsymbol{i}\boldsymbol{,}\boldsymbol{j}}\text{ and }\boldsymbol{H}_{\boldsymbol{i}\boldsymbol{,}\boldsymbol{max}}$is the maximal value of $\boldsymbol{H}_{\boldsymbol{i}\boldsymbol{,}\boldsymbol{j}}$. $\boldsymbol{\alpha}$ is a weight parameter ($\boldsymbol{\alpha}\boldsymbol{\in[0,1]}$). The H criterion gain is expressed as a percentage of the initial value of H calculated using the actual recipient x donor crosses for each recipient.

# Supplemental tables

Table S1 Estimation of the mean performance (μ) and additive genetic variance ($\boldsymbol{\sigma}_{\mathbf{A}}^{\mathbf{2}}$) of the observed hybrid families.


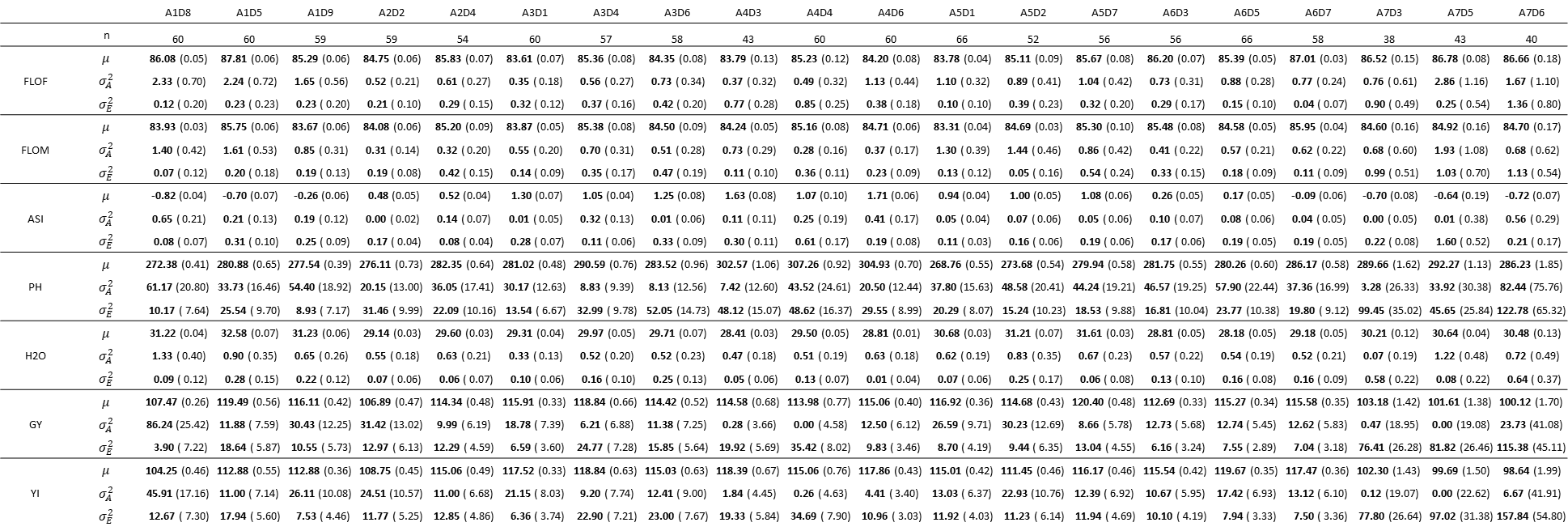
Estimations were performed with a mixed linear model independently in each family. $\boldsymbol{n}$: number of individuals, $\boldsymbol{\sigma}_{\boldsymbol{E}}^{\boldsymbol{2}}$ : error variance. Standard errors are indicated in brackets.


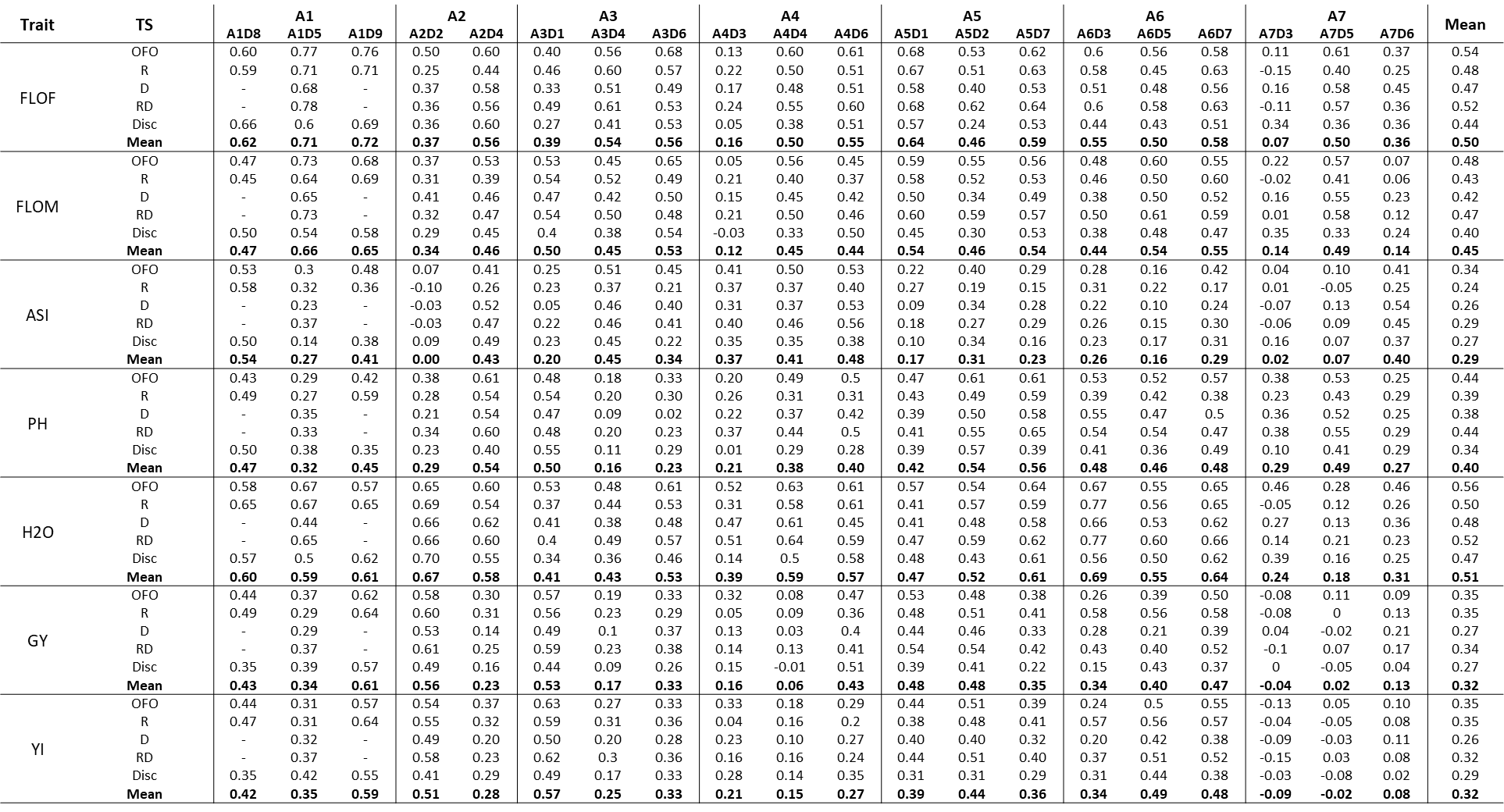
Table S2 Predictive abilities for the prediction of the hybrid values of the observed families using different training sets.

For each family, two-thirds of the hybrids were sampled for use as a validation set and their values were predicted thanks to a GBLUP model calibrated with different TS types (F, OFO, R, RD or Disc). One hundred repetitions were performed.


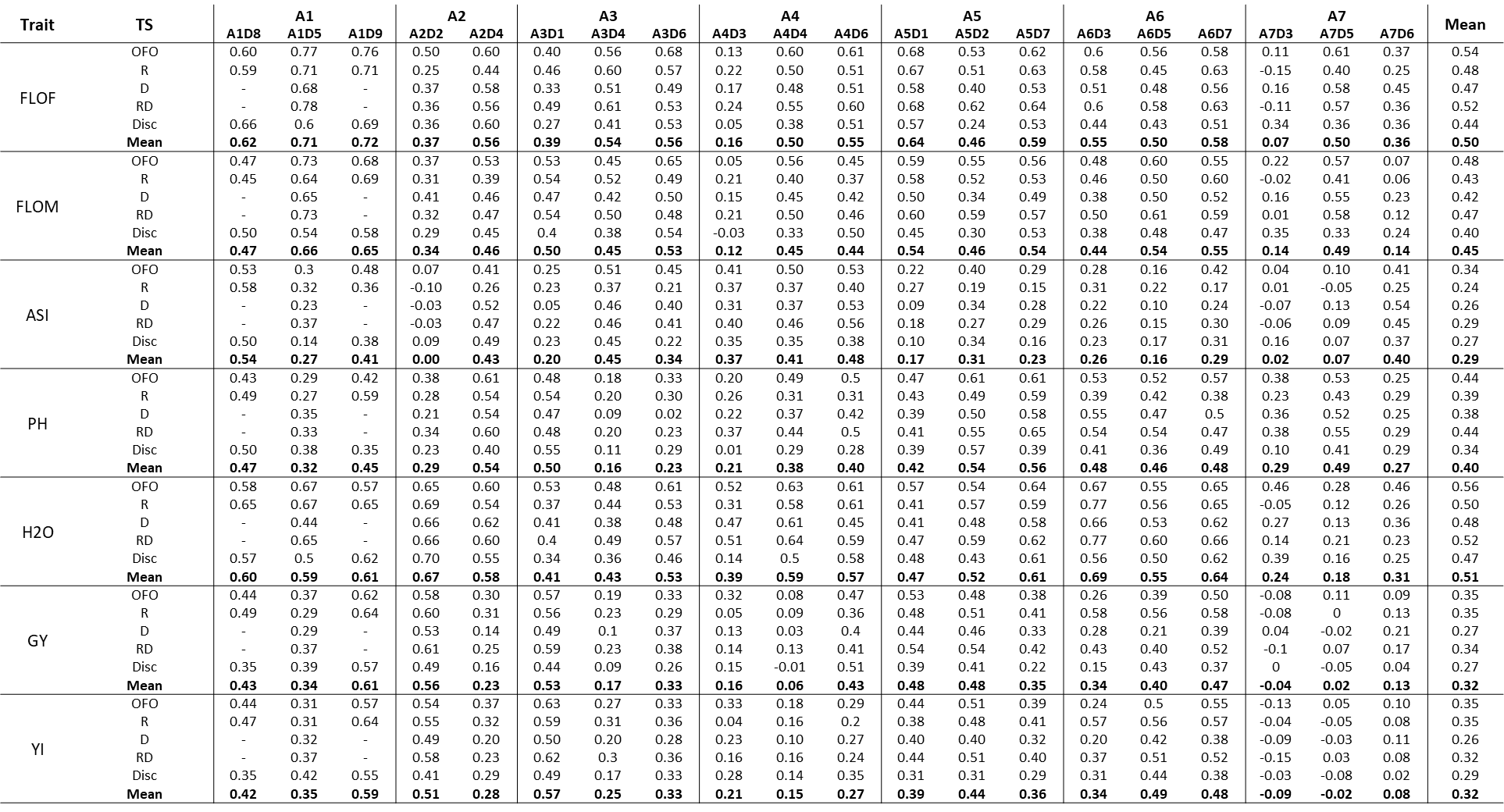
Table S3 Predictive abilities for the prediction of the hybrid values of the observed families using different training sets (addition of individuals derived from the predicted cross in the TS).

For each family, two-thirds of the hybrids were sampled for use as a validation set. Their values were predicted thanks to a GBLUP model calibrated with different TS types (F, OFO, R, RD or Disc) completed with the remaining third. One hundred repetitions were performed.


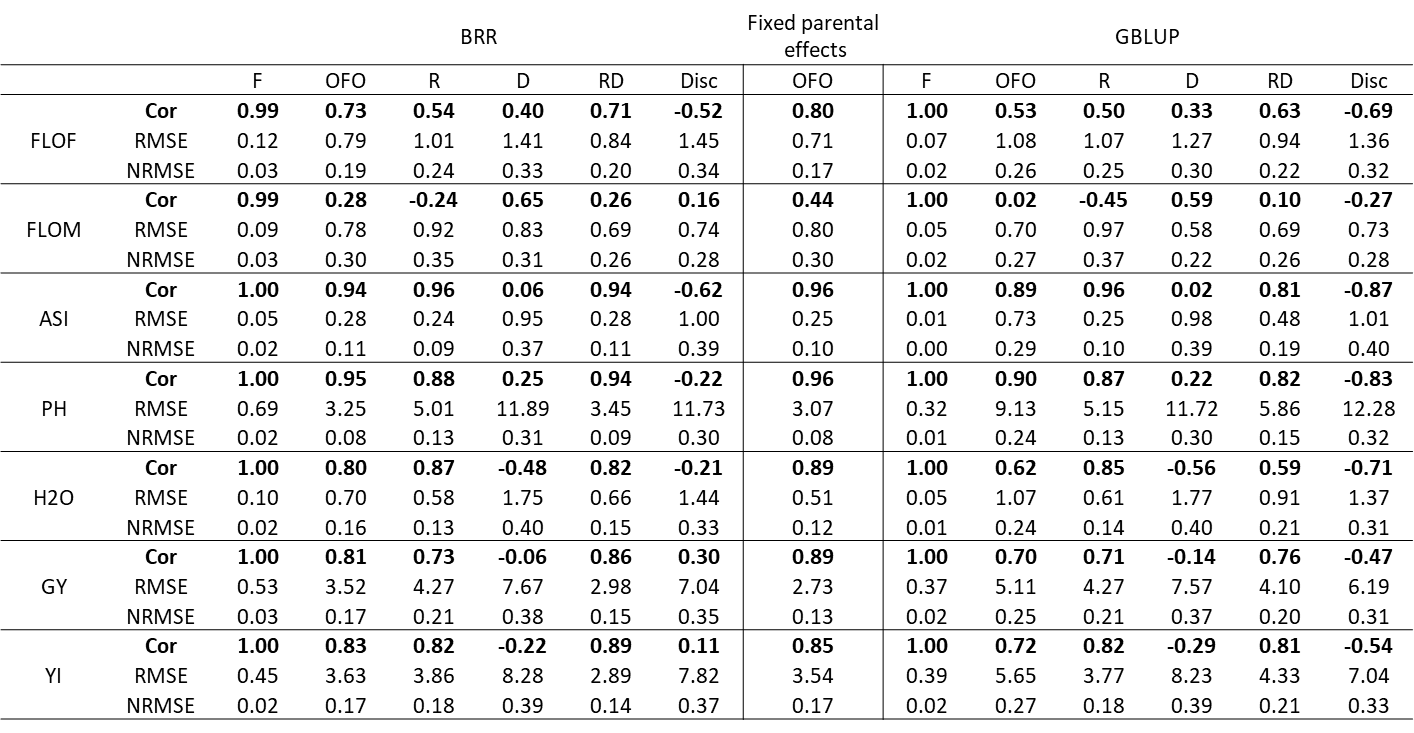
Table S4 Correlations, Root Mean Square Errors (RMSE) and Normalized RMSE (NRMSEs) obtained for the prediction of the mean performances of the observed cross.

BRR: For a cross $\boldsymbol{i \in[1,20]}$ and a training set type $\boldsymbol{j \in\{}\boldsymbol{F, OFO, R, D, RD, Disc\}}$, marker effects were estimated with a Bayesian Ridge Regression model calibrated with $\boldsymbol{j}$to predict the mean performance of $\boldsymbol{i}$. GBLUP: Same process using marker effects backsolved from a GBLUP model. Fixed parental effect: For a cross $\boldsymbol{i \in[1,20]}$, mean performances were predicted from the estimation of fixed parental effects with the training set type OFO. We computed the correlation between the correlation between the observed ($\boldsymbol{\mu}_{\boldsymbol{obs}\boldsymbol{,}\boldsymbol{i}}$) and predicted ($\boldsymbol{\mu}_{\boldsymbol{pred}\boldsymbol{,}\boldsymbol{i}}$) mean performances over twenty crosses. $\boldsymbol{RMSE=}\sqrt{\frac{\boldsymbol{1}}{\boldsymbol{20}}\sum_{\boldsymbol{i}\boldsymbol{=1}}^{\boldsymbol{20}} \left( \boldsymbol{\mu}_{\boldsymbol{pred}\boldsymbol{,}\boldsymbol{i}}\boldsymbol{-}\boldsymbol{\mu}_{\boldsymbol{obs}\boldsymbol{,}\boldsymbol{i}} \right)^{\boldsymbol{2}}}$. $\boldsymbol{NRMSE}\boldsymbol{=}\boldsymbol{RMSE}\boldsymbol{/}\left( \boldsymbol{\mu}_{\boldsymbol{obs,max}}\boldsymbol{-}\boldsymbol{\mu}_{\boldsymbol{obs,min}} \right)$ where $\boldsymbol{\mu}_{\boldsymbol{obs,min}}$ and $\boldsymbol{\mu}_{\boldsymbol{obs,min}}$ indicate the minimum and maximum means observed across families, respectively.


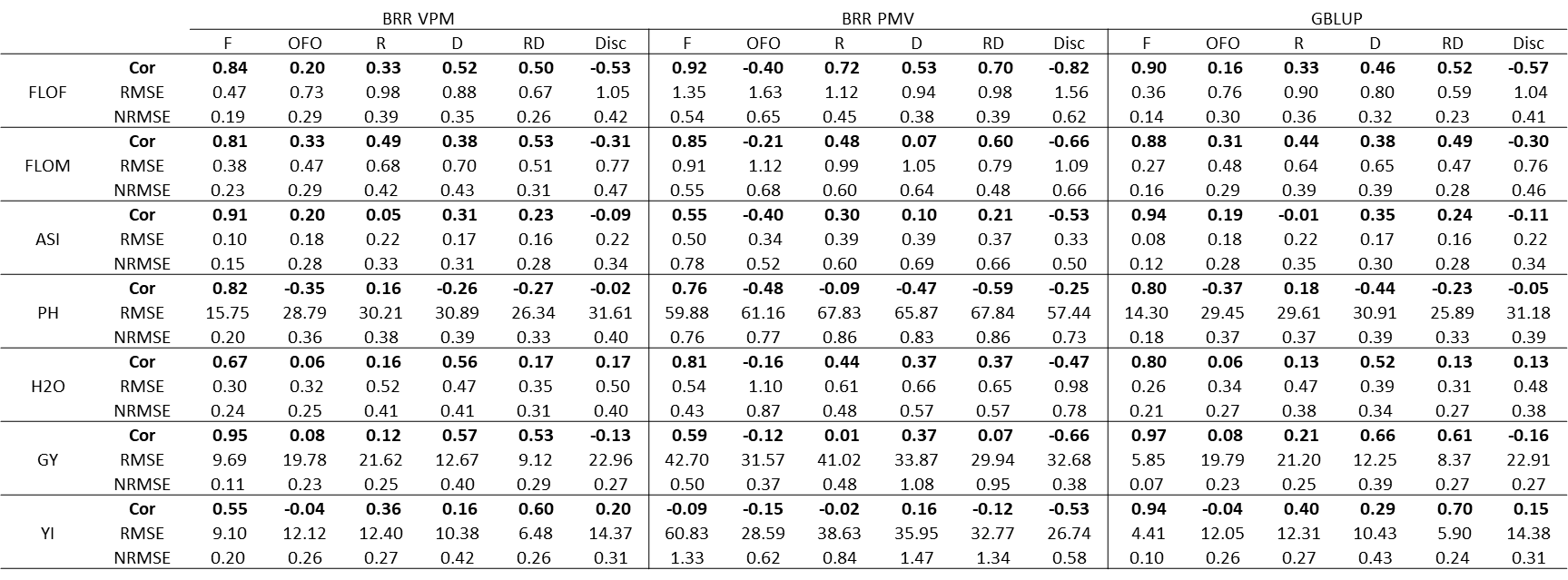
Table S5 Correlations, Root Mean Square Errors (RMSE) and Normalized RMSE (NRMSEs) obtained for the prediction of the variance of the observed cross.

BRR: For a cross $\boldsymbol{i \in[1,20]}$ and a training set type $\boldsymbol{j \in\{}\boldsymbol{F, OFO, R, D, RD, Disc\}}$, marker effects were estimated with a Bayesian Ridge Regression model calibrated with $\boldsymbol{j}$to predict the variance of $\boldsymbol{i}$. GBLUP: Same process using marker effects backsolved from a GBLUP model. For BRR, two variance prediction methods were compared: “variance of the posterior means” (*VPM*) and “posterior mean variance” (*PMV*). We computed the correlation between the correlation between the observed ($\boldsymbol{\sigma}_{\boldsymbol{A}\boldsymbol{,}\boldsymbol{obs}\boldsymbol{,}\boldsymbol{i}}^{\boldsymbol{2}}$) and predicted ($\boldsymbol{\sigma}_{\boldsymbol{A}\boldsymbol{,}\boldsymbol{pred}\boldsymbol{,}\boldsymbol{i}}^{\boldsymbol{2}}$) variances over twenty crosses. $\boldsymbol{RMSE=}\sqrt{\frac{\boldsymbol{1}}{\boldsymbol{20}}\sum_{\boldsymbol{i}\boldsymbol{=1}}^{\boldsymbol{20}} \left( \boldsymbol{\sigma}_{\boldsymbol{A}\boldsymbol{,}\boldsymbol{pred}\boldsymbol{,}\boldsymbol{i}}^{\boldsymbol{2}}\boldsymbol{-}\boldsymbol{\sigma}_{\boldsymbol{A}\boldsymbol{,}\boldsymbol{obs}\boldsymbol{,}\boldsymbol{i}}^{\boldsymbol{2}} \right)^{\boldsymbol{2}}}$. $\boldsymbol{NRMSE}\boldsymbol{=}\boldsymbol{RMSE}\boldsymbol{/}\left( \boldsymbol{\sigma}_{\boldsymbol{A}\boldsymbol{,}\boldsymbol{obs}\boldsymbol{,}\boldsymbol{max}}^{\boldsymbol{2}}\boldsymbol{-}\boldsymbol{\sigma}_{\boldsymbol{A}\boldsymbol{,}\boldsymbol{obs}\boldsymbol{,}\boldsymbol{min}}^{\boldsymbol{2}} \right)$ where $\boldsymbol{\sigma}_{\boldsymbol{A}\boldsymbol{,}\boldsymbol{obs}\boldsymbol{,}\boldsymbol{min}}^{\boldsymbol{2}}$ and $\boldsymbol{\sigma}_{\boldsymbol{A}\boldsymbol{,}\boldsymbol{obs}\boldsymbol{,}\boldsymbol{max}}^{\boldsymbol{2}}$ indicate the minimum and maximum variance observed across families, respectively.

**Table S6 Computation and composition of the usefulness criterion (UC) for GY and YI**

|  |  | A1D2 | A1D5 | A1D7 | A2D2 | A2D4 | A3D1 | A3D4 | A3D6 | A4D3 | A4D4 | A4D6 | A5D1 | A5D2 | A5D7 | A6D3 | A6D5 | A6D7 | A7D3 | A7D5 | A7D6 |
| --- | --- | --- | --- | --- | --- | --- | --- | --- | --- | --- | --- | --- | --- | --- | --- | --- | --- | --- | --- | --- | --- |
|  | n | 60 | 60 | 59 | 59 | 54 | 60 | 57 | 58 | 43 | 60 | 60 | 66 | 52 | 56 | 56 | 66 | 58 | 38 | 43 | 40 |
| GY | $\mu$ | 107.47 | 119.49 | 116.11 | 106.89 | 114.34 | 115.91 | 118.84 | 114.42 | 114.58 | 113.98 | 115.06 | 116.92 | 114.68 | 120.4 | 112.69 | 115.27 | 115.58 | 103.18 | 101.61 | 100.12 |
|  | $\alpha_{A}^{2}$ | 86.24 | 11.88 | 30.43 | 31.42 | 9.99 | 18.78 | 6.21 | 11.38 | 0.28 | 0 | 12.5 | 26.59 | 30.23 | 8.66 | 12.73 | 12.74 | 12.62 | 0.47 | 0 | 23.73 |
|  | $UC$ | **126.69** | **126.62** | **127.53** | **118.49** | **120.88** | **124.88** | **124.00** | **121.40** | **115.68** | **113.98** | **122.38** | **127.59** | **126.06** | **126.49** | **120.08** | **122.66** | **122.93** | **104.60** | **101.61** | **110.20** |
| YI | $\mu$ | 104.25 | 112.88 | 112.88 | 108.75 | 115.06 | 117.52 | 118.84 | 115.03 | 118.39 | 115.06 | 117.86 | 115.01 | 111.45 | 116.17 | 115.54 | 119.67 | 117.47 | 102.3 | 99.69 | 98.64 |
|  | $\alpha_{A}^{2}$ | 45.91 | 11 | 26.11 | 24.51 | 11 | 21.15 | 9.2 | 12.41 | 1.84 | 0.26 | 4.41 | 13.03 | 22.93 | 12.39 | 10.67 | 17.42 | 13.12 | 0.12 | 0 | 6.67 |
|  | $UC$ | **118.28** | **119.75** | **123.46** | **119.00** | **121.93** | **127.04** | **125.12** | **122.32** | **121.20** | **116.12** | **122.21** | **122.48** | **121.36** | **123.46** | **122.30** | **128.31** | **124.97** | **103.02** | **99.69** | **103.99** |

For each cross and each trait, the usefulness criterion value was computed using the mean performance ($\mu$) and the additive variance ($\sigma_{A}^{2}$) estimated with a mixed linear model ($M1$) as $UC=\mu+i* \sigma_{a}$ where $i$ is the intensity selection (we took $i=2.07.$ corresponding to a selection rate of 5%).

Table S7 Predicted usefulness criterion for the not observed crosses in our experimental design between the recipient lines (in rows) and the donor lines (in columns)

|  | **D1** | **D2** | **D3** | **D4** | **D5** | **D6** | **D7** | **D8** | **D9** |
| --- | --- | --- | --- | --- | --- | --- | --- | --- | --- |
| **A1** | 127.04 | 126.01 | 123.99 | 125.08 | - | 125.03 | 125.14 | - | - |
| **A2** | 119.23 | - | 118.02 | - | 118.84 | 119.74 | 121.08 | 119.31 | 118.65 |
| **A3** | - | 123.88 | 121.49 | - | 121.68 | - | 123.17 | 122.02 | 122.27 |
| **A4** | 120.35 | 121.48 | - | - | 118.49 | - | 119.14 | 118.70 | 118.38 |
| **A5** | - | - | 124.71 | 124.53 | 124.64 | 125.37 | - | 126.92 | 125.93 |
| **A6** | 123.36 | 124.44 | - | 119.52 | - | 122.55 | - | 120.64 | 122.04 |
| **A7** | 106.45 | 105.63 | - | 106.90 | - | - | 106.14 | 104.31 | 104.61 |

These predictions were obtained using a Bayesian Ridge Regression model calibrated with specific training set for each predicted cross (RD type) following the VPM method.

Table S8 Evolution of the H criterion for each recipient line during forward selection of new donor lines based on H criterion or the usefulness criterion of the incorporated donors ($\bar{\boldsymbol{UC}}$).

| **Number of incorporated donors** | **1** | | **2** | | **3** | | **4** | | **5** | | **6** | | **7** | |
| --- | --- | --- | --- | --- | --- | --- | --- | --- | --- | --- | --- | --- | --- | --- |
| **Selection Index**  **Recipient line** | **H** | $\bar{\boldsymbol{UC}}$ | **H** | $\bar{\boldsymbol{UC}}$ | **H** | $\bar{\boldsymbol{UC}}$ | **H** | $\bar{\boldsymbol{UC}}$ | **H** | $\bar{\boldsymbol{UC}}$ | **H** | $\bar{\boldsymbol{UC}}$ | **H** | $\bar{\boldsymbol{UC}}$ |
| **A1** | 9.99 | 8.00 | 16.70 | 11.22 | 20.67 | 16.20 | 23.30 | 20.60 | 24.71 | 24.62 | 25.95 | 25.95 | - | - |
| **A2** | 25.71 | 13.91 | 33.38 | 26.88 | 37.62 | 29.07 | 40.22 | 34.16 | 42.29 | 40.73 | 43.25 | 42.75 | 43.82 | 43.82 |
| **A3** | 14.60 | 6.57 | 18.70 | 11.89 | 21.07 | 15.31 | 22.86 | 17.24 | 24.10 | 22.92 | 24.86 | 24.86 | - | - |
| **A4** | 16.96 | 8.55 | 22.56 | 15.98 | 25.26 | 19.49 | 27.16 | 20.59 | 28.78 | 27.06 | 29.22 | 29.22 | - | - |
| **A5** | 13.28 | 3.63 | 18.77 | 9.60 | 21.81 | 13.65 | 24.03 | 16.25 | 25.47 | 22.42 | 26.00 | 26.00 | - | - |
| **A6** | 10.92 | 8.20 | 17.05 | 14.69 | 21.64 | 20.26 | 24.58 | 23.09 | 26.85 | 24.01 | 27.58 | 27.58 | - | - |
| **A7** | 12.66 | 12.66 | 19.25 | 19.25 | 22.12 | 20.86 | 24.45 | 23.61 | 25.79 | 25.79 | 26.60 | 26.60 | - | - |

The H criterion gain is expressed as a percentage of the value of H calculated using the initial recipient x donor crosses for each recipient. Note that the A2 recipient was crossed to only two donors instead of three, leading to seven donors remaining to be incorporated instead of six.

Table S9 Evolution of the mean usefulness criterion values of the incorporated donors ($\bar{\boldsymbol{UC}}$) during forward selection of new donor lines based on the H criterion or the $\bar{\boldsymbol{UC}}$.

| **Number of incorporated donors** | **1** | | **2** | | **3** | | **4** | | **5** | | **6** | | **7** | |
| --- | --- | --- | --- | --- | --- | --- | --- | --- | --- | --- | --- | --- | --- | --- |
| **Selection Index**  **Recipient line** | **H** | $\bar{\boldsymbol{UC}}$ | **H** | $\bar{\boldsymbol{UC}}$ | **H** | $\bar{\boldsymbol{UC}}$ | **H** | $\bar{\boldsymbol{UC}}$ | **H** | $\bar{\boldsymbol{UC}}$ | **H** | $\bar{\boldsymbol{UC}}$ | **H** | $\bar{\boldsymbol{UC}}$ |
| **A1** | 125.03 | 127.04 | 125.06 | 126.52 | 125.72 | 126.06 | 125.28 | 125.82 | 125.43 | 125.66 | 125.38 | 125.38 | - | - |
| **A2** | 118.84 | 121.08 | 119.29 | 120.41 | 119.27 | 120.04 | 118.96 | 119.84 | 118.90 | 119.64 | 119.26 | 119.47 | 119.27 | 119.27 |
| **A3** | 121.68 | 123.88 | 121.59 | 123.53 | 121.82 | 123.11 | 122.33 | 122.84 | 122.50 | 122.61 | 122.42 | 122.42 | - | - |
| **A4** | 118.49 | 121.48 | 119.42 | 120.91 | 119.07 | 120.32 | 119.09 | 119.92 | 119.57 | 119.63 | 119.42 | 119.42 | - | - |
| **A5** | 124.64 | 126.92 | 124.59 | 126.43 | 124.85 | 126.07 | 125.12 | 125.73 | 125.04 | 125.52 | 125.35 | 125.35 | - | - |
| **A6** | 122.55 | 124.44 | 121.04 | 123.90 | 121.81 | 123.45 | 122.47 | 123.10 | 122.38 | 122.61 | 122.09 | 122.09 | - | - |
| **A7** | 106.90 | 106.90 | 106.67 | 106.67 | 106.33 | 106.50 | 105.90 | 106.28 | 105.95 | 105.95 | 105.67 | 105.67 | - | - |

The H criterion gain is expressed as a percentage of the value of H calculated using the initial recipient x donor crosses for each recipient. Note that the A2 recipient was crossed to only two donors instead of three, leading to seven donors remaining to be incorporated instead of six.
